# A *Francisella novicida* Mutant, Lacking the Soluble Lytic Transglycosylase Slt, Exhibits Defects in Both Growth and Virulence

**DOI:** 10.3389/fmicb.2019.01343

**Published:** 2019-06-14

**Authors:** Beth A. Bachert, Sergei S. Biryukov, Jennifer Chua, Sabrina A. Rodriguez, Ronald G. Toothman, Christopher K. Cote, Christopher P. Klimko, Melissa Hunter, Jennifer L. Shoe, Janice A. Williams, Kathleen A. Kuehl, Fabrice V. Biot, Joel A. Bozue

**Affiliations:** ^1^Bacteriology Division, United States Army Medical Research Institute of Infectious Diseases (USAMRIID), Frederick, MD, United States; ^2^Pathology Division, United States Army Medical Research Institute of Infectious Diseases (USAMRIID), Frederick, MD, United States; ^3^Unité de Bactériologie/UMR_MD1, Département de Biologie des Agents Transmissibles, Institut de Recherche Biomédicale des Armées, Brétigny-sur-Orge, France

**Keywords:** *Francisella*, peptidoglycan (PG), tularemia, *Francisella novicida*, virulence, cell morphology, lytic transglycosylase, cell division

## Abstract

*Francisella tularensis* is the causative agent of tularemia and has gained recent interest as it poses a significant biothreat risk. *F. novicida* is commonly used as a laboratory surrogate for tularemia research due to genetic similarity and susceptibility of mice to infection. Currently, there is no FDA-approved tularemia vaccine, and identifying therapeutic targets remains a critical gap in strategies for combating this pathogen. Here, we investigate the soluble lytic transglycosylase or Slt in *F. novicida*, which belongs to a class of peptidoglycan-modifying enzymes known to be involved in cell division. We assess the role of Slt in biology and virulence of the organism as well as the vaccine potential of the *slt* mutant. We show that the *F. novicida slt* mutant has a significant growth defect in acidic pH conditions. Further microscopic analysis revealed significantly altered cell morphology compared to wild-type, including larger cell size, extensive membrane protrusions, and cell clumping and fusion, which was partially restored by growth in neutral pH or genetic complementation. Viability of the mutant was also significantly decreased during growth in acidic medium, but not at neutral pH. Furthermore, the *slt* mutant exhibited significant attenuation in a murine model of intranasal infection and virulence could be restored by genetic complementation. Moreover, we could protect mice using the *slt* mutant as a live vaccine strain against challenge with the parent strain; however, we were not able to protect against challenge with the fully virulent *F. tularensis* Schu S4 strain. These studies demonstrate a critical role for the Slt enzyme in maintaining proper cell division and morphology in acidic conditions, as well as replication and virulence *in vivo*. Our results suggest that although the current vaccination strategy with *F. novicida slt* mutant would not protect against Schu S4 challenges, the Slt enzyme could be an ideal target for future therapeutic development.

## Introduction

*Francisella tularensis* is a Gram-negative coccobacillus which causes the disease tularemia, or “rabbit fever” in humans. Rabbits and several rodents are the primary reservoir, but this organism has also been found in a wide range of mammals and birds. Infection mainly occurs via the bite of an infected tick, deerfly, or mosquito, resulting in the ulceroglandular form of disease. *Francisella* primarily infects macrophages, which are thought to be the major reservoir for the bacteria *in vivo* and critical for its ability to cause disease in animals and humans (McLendon et al., 2006). However, *Francisella* has been shown to replicate in other cell types, including human lung epithelial cells, dendritic cells, neutrophils, fibroblasts, and hepatocytes (Ben Nasr et al., 2006; Melillo et al., 2006; Qin et al., 2009; Horzempa et al., 2010; Law et al., 2011; Schwartz et al., 2012; Bradburne et al., 2013).

*Francisella tularensis* is comprised of two subspecies that cause disease in humans, *tularensis* (type A) and *holarctica* (type B). *F. tularensis* subsp. *tularensis* is highly virulent and causes disease primarily in North America, while *F. tularensis* subsp. *holarctica* is common throughout the Northern hemisphere and causes a less severe disease (Ellis et al., 2002). A third subspecies, *novicida*, rarely causes disease in humans, but is commonly used as a surrogate strain for fully virulent *F. tularensis*. It has a high degree of genetic similarity, can infect macrophages, produces disease in mice, and can be handled under BSL-2 conditions (Kingry and Petersen, 2014).

The Centers for Disease Control and Prevention reported approximately 1,800 cases of tularemia in the United States during 2006 to 2016, most occurring in the mid-western and northeastern states^1^. While the overall incidence of naturally acquired tularemia cases is low, recent decades have seen a renewed interest in *F. tularensis* due to its potential to be used as a bioweapon. The bacteria can be easily obtained from the environment and aerosolized, has an infectious dose reported to be as low as a single bacterium, and no Food and Drug Administration (FDA)-approved vaccine is available (Jones et al., 2005; Kingry and Petersen, 2014). If left untreated, tularemia has a mortality rate of up to 60% (McCrumb, 1961). Tularemia has been traditionally treated through antibiotic therapy with streptomycin, doxycycline, or ciprofloxacin, although newly available antibiotics such as ketolids, tigecycline, and fluoroquinolones are currently being evaluated for treatment (Boisset et al., 2014). The development of antibiotic resistance in *Francisella*, either naturally acquired or purposely engineered, is a significant concern from a biodefense standpoint. The latter has been frequently described in the literature (Gestin et al., 2010; Loveless et al., 2010; Sutera et al., 2014).

A live vaccine strain (LVS), derived from *F. holarctica*, was developed by the former Soviet Union. In the United States, the LVS vaccine is only available as an investigational vaccine (Oyston and Quarry, 2005). Additionally, there are several concerns about LVS which has hindered its licensure by the FDA. These concerns include potential reversion of the strain back to a virulent state, as the exact basis for loss of virulence is unknown, and possible breakthrough in protection (Eigelsbach and Downs, 1961; Saslaw et al., 1961; Hornick and Eigelsbach, 1966). However, for intracellular pathogens such as *Francisella*, live-attenuated vaccines could potentially provide better protection than a subunit vaccine. Live attenuated vaccines establish mild infections in the host, mimicking the infection of fully virulent wild-type strains and presenting appropriate antigens to the host immune system to induce durable antibody and cell-mediated immune responses (Drabner and Guzman, 2001). Genetically engineered bacterial strains can have well-defined mechanisms of attenuation, making them safer, better-characterized alternatives as candidate live vaccine strains (Bozue et al., 2012). For instance, numerous deletion mutants have been generated in *Francisella* strains and tested as live vaccine candidates to protect against tularemia. Several defined mutants of *Francisella* have shown promise for protection in several tularemia models (Qin et al., 2009, 2011; Twine et al., 2012; Golovliov et al., 2013; Chu et al., 2014; Jia and Horwitz, 2018). While significant progress has been made in understanding the virulence of this organism, additional efforts are needed to identify novel targets, such as cell replicative enzymes, for development of medical countermeasures.

Cell division in Gram-negative bacteria is accomplished through the coordinated actions of penicillin binding proteins (PBPs) and other peptidoglycan-modifying enzymes. These enzymes harbor functions including transpeptidase, carboxypeptidase, lysozyme, and lytic transglycosylase activities. Redundancy of one or more of these enzymes is commonly observed in many bacterial species, i.e., multiple PBPs in a single organism. The lytic transglycosylases (LTs) are a class of enzymes which recycle peptidoglycan via cleavage of the β-1,4-glycosidic bond between *N*-acetylglucosamine and *N*-acetylmuramic acid residues, while also forming glycosidic bonds with the C6 hydroxyl group of the muramic acid (Dijkstra and Thunnissen, 1994; Scheurwater et al., 2008). These activities allow elongation and separation of the daughter cells. Several reviews highlight the role of LTs in allowing expansion of the sacculus and septum splitting to form daughter cells, peptidoglycan turnover, and also incorporation of pili and secretion systems (Dijkstra and Thunnissen, 1994; Scheurwater et al., 2008; Dik et al., 2017). Moreover, peptidoglycan-modifying enzymes have been recommended as promising targets for antibiotics (Liu and Breukink, 2016).

Given the importance of LTs in maintaining cell structure and proper cell division, as well as its potential for antimicrobial targeting, we investigated the importance of the soluble lytic transglycosylase (Slt) in *Francisella*. Initial efforts in our laboratory were focused on mutating the *slt* gene in LVS and *F. tularensis* SchuS4 strains. Strategies including Targetron-based mutagenesis and in-frame deletion via suicide plasmids were unsuccessful in both LVS and SchuS4, indicating *slt* is likely essential for survival in these organisms. Therefore, in order to assess the function of Slt, we utilized an *F. novicida slt* mutant isolated from an existing transposon library (Gallagher et al., 2007). We show that the loss of the *slt* gene in *F. novicida* significantly affected growth, viability, and cell morphology in a pH-dependent manner, demonstrating its importance in maintaining cell physiology. We found that inactivation of *slt* resulted in significantly attenuated virulence in both macrophages and mice. Moreover, we show that vaccination of mice with the mutant strain protected against challenge with the wild-type (WT) *F. novicida* strain but not against challenge with the *F. tularensis* Schu S4 strain. Overall, we demonstrate the impact of this enzyme in the biology and virulence of *Francisella*.

## Materials and Methods

### Bacterial Strains

All strains and plasmids used in this study are listed in Table 1. *Escherichia coli* NEB Turbo cells (New England Biolabs, Ipswich, MA, United States) were used for cloning purposes. *E. coli* was propagated in Luria-Bertani broth or agar supplemented with ampicillin at 100 μg/ml, hygromycin at 200 μg/ml, or kanamycin at 20 μg/ml as necessary. All cultures were grown at 37°C.

**Table 1:** Strains and plasmids used in this study.

		Source
***E. coli***		
NEB Turbo	Cloning strain	NEB
***F. novicida***		
U112 strain	*F. tularensis* subsp. *novicida*	ATCC 15482 (Ellis et al., 2002)
*slt*::T20	Inactivated *slt* (BEI catalog # NR-6640)	BEI (Gallagher et al., 2007)
*slt*::T20 with pMP822+*slt*	Complemented *slt*::T20	This study
***F. tularensis***		
Schu S4	Fully virulent Type A strain	USAMRIID collection
***F. holarctica***		
LVS	Live vaccine strain	USAMRIID collection
**Plasmids**		
pMP822	Complementation plasmid	LoVullo et al., 2009
pMP822+*slt*	Plasmid containing the intact Fn *slt* gene	This study

*Francisella tularensis* subsp. *tularensis* Schu S4 and LVS, derived from *F. tularensis* subsp. *holarctica*, were utilized for initial mutagenesis studies. *F. tularensis* subsp. *novicida* strain U112 and transposon derivatives (Gallagher et al., 2007) (BEI) were used in this study (Table 1). *F. novicida* transposon mutants NR-6234 and NR-6640 have T20 inserted into the *slt* ORF at bp position 930 or 479, respectively. The *F. novicida slt* gene is 1977 bps in length, therefore the strain with the transposon inserted closer to the 5′ end was pursued for further studies. For routine growth of *F. tularensis* species, bacteria were grown on enriched chocolate agar plates obtained from Remel^TM^ (product number R01300; Lenexa, KS, United States). When necessary, agar was supplemented with kanamycin at 10 μg/ml and/or hygromycin at 200 μg/ml. As indicated, *F. novicida* was grown in broth culture in Chamberlain’s Defined Medium (CDM) (Chamberlain, 1965) or brain heart infusion (BHI) supplemented with 1% IsoVitaleX^TM^ (Becton Dickinson, Cockeysville, MD, United States).

### Mutagenesis

Mutagenesis studies were carried out utilizing the LVS and *F. tularensis* Schu S4 strains. Two plasmid constructs were designed to generate interruptions at positions 267 or 418 within the *slt* open reading frame (ORF), respectively, via group II intron incorporation using the Targetron gene knockout system, as previously described (Rodriguez et al., 2008). *Francisella* strains were grown to mid-logarithmic phase in BHI supplemented with 1% IsoVitaleX, then thoroughly washed with 0.5 M sucrose. Electroporations were carried out using a Bio-Rad Gene Pulser Xcell for 2.5 kV, 25 μF, and 600 Ω, followed by recovery in BHI with IsoVitaleX for up to 3 h at 30°C and 200 rpm. Transformants were selected on chocolate agar plates containing kanamycin. Intron and gene-specific primer pairs were used in PCR to screen for insertions, and colonies containing both WT (wild-type) and mutant alleles were passaged at 30°C and re-screened for loss of the WT allele.

In-frame deletion of the *slt* allele was attempted using pMP590, a *Francisella* suicide vector containing the *sacB* counterselectable marker, modified to include a 1,680 bp deletion in *slt*, with approximately 1 kb flanking homologous sequences for recombination (LoVullo et al., 2006). Electroporation was carried out as described above, with the exception that cells were recovered and grown at 37°C. Kanamycin-resistant colonies were further passaged onto chocolate agar containing up to 8% sucrose to promote the second recombination event and resolution of the WT allele. PCR screening was performed to determine the presence of WT and mutant alleles.

### Complementation

To demonstrate that observed phenotypes observed for the *F. novicida slt* mutant was due specifically to inactivation of this gene, the functional *slt* gene was PCR amplified and cloned into shuttle vector pMP822 (LoVullo et al., 2009), using primers AW25_RS01533_compF (5′- acacggtccgttaagctaaatatactaatttttgcg) and AW21_RS06205_ compR (5′- aatcccgggatcaaatatgtagataatctcagcc) containing RsrII and SmaI sites, respectively. Plasmid sequence was confirmed by Sanger sequencing. The *slt* mutant strain was transformed with pMP822+*slt* via electroporation as described above (LoVullo et al., 2006). Cells were recovered for up to 3 h at 37°C and 200 rpm, and transformants were selected on chocolate agar plates containing both kanamycin and hygromycin.

### Growth Curves

Growth assays were performed in CDM adjusted to pH 5.0, 6.2, or 7.0, or in BHI broth supplemented with 1% IsoVitaleX. Assays were performed using an Infinite M200 Pro (Tecan; Männedorf, Switzerland) microplate reader in 96-well microtiter plates at 37°C with shaking. The OD_600_ was measured every 60 min. For all assays, *F. novicida* strains were grown for 18 h on chocolate agar plates and then resuspended in broth medium to an equal OD_600_. Three independent growth assays were performed, with quadruplicate wells for each strain. Medium controls were included to confirm sterility and for use as blanks to calculate the absorbance of the cultures.

### Scanning Electron Microscopy (SEM)

*Francisella novicida* strains were grown in CDM to mid-logarithmic phase (OD_600_ ∼0.5), and fixed with 1% glutaraldehyde and 4% formaldehyde in cacodylate buffer. A droplet of each bacteria was applied to a poly-L lysine coated glass coverslip for 5 min at room temperature to allow for bacteria to attach to the coverslip and briefly fixed with 2% glutaraldehyde. After, the samples were rinsed in cacodylate buffer, post fixed for 1 h at room temperature in cacodylate buffer containing 1% osmium tetroxide, stained with ethanolic uranyl acetate, and dehydrated in a graded series of ethanol. Samples were rinsed at least three times in 100% ethanol before being critically point dried. Samples were then mounted on aluminum stubs, sputter coated with platinum and then imaged in the Zeiss Sigma VP scanning electron microscope at various magnifications. Preparation of bacteria and SEM analysis was performed from two independent bacterial cultures for each strain, and yielded similar results.

### Live/Dead Assay and Confocal Microscopy

*Francisella novicida* strains were grown for 18 h on chocolate agar plates and resuspended in CDM adjusted to either pH 5.0 or 7.0 to an OD_600_ of ∼0.5. A volume of 1 mL was removed for initial viability assessment, and the remaining cell suspension was used to inoculate CDM to a starting OD_600_ of ∼0.05. Cultures were grown to mid-logarithmic phase, ∼6 h, and cell staining was performed using the LIVE/DEAD^®^ *Bac*Light^TM^ Bacterial Viability kit (Life Technologies, Grand Island, NY, United States) in accordance with the manufacturer’s protocol. Briefly, bacteria were incubated in 0.17 mM Syto 9 and 1.8 mM propidium iodide (PI) for 5 min prior to imaging. Fluorescent and differential interference contrast (DIC) images were taken on the Zeiss 700 Laser Scanning Confocal Microscope System using a 100 × 1.4 numerical aperture oil objectives lens with the pinhole set to 1 Airy unit. Dead bacteria counts include those that stained with PI and lysed bacteria that do not stain with either dye. Images were collected from three independent experiments, wherein a range of 100–500 cells were counted per sample.

### *In vitro* Susceptibility Assays

The minimal inhibitory concentrations (MICs) for polymyxin B were determined by the *E*-test procedure (Biomerieux, Inc., Marcy-l’Étoile, France) on chocolate agar. Results were read after incubation for 24 h at 37°C and are expressed in mg/L. Sensitivity of strains to Tween 20, Triton X-100, sodium dodecyl sulfate, and sodium deoxycholate were tested via disk diffusion, and zone of inhibition was measured after incubation for 24 h at 37°C. Results are representative of at least three independent experiments where three *E*-tests or disks were tested for each strain.

### Macrophage Assays

J774A.1 cells, a murine macrophage-like cell line obtained from the American Type Culture Collection, were seeded (∼2.5 × 10^5^ cells/well) into 24-well plates and cultured 24 h (37°C, 5% CO_2_) at which time the cells had formed confluent monolayers. The cells were maintained in Dulbecco’s Modified Eagle’s medium (D-MEM, Corning #10-013-CV) with 4.5 g/L glucose, L-glutamine, and sodium pyruvate, supplemented with 10% heat-inactivated fetal bovine serum (FBS) and additional L-glutamine (HyClone, #SH30034.01). For the intracellular assays, *F. novicida* was suspended in phosphate buffered saline (PBS) from an 18 h plate, respectively, and then diluted 1:5 in tissue culture medium. 200 μL of the bacterial suspension was added to the macrophages to achieve a multiplicity of infection (MOI) of ∼100:1, and the MOI was confirmed from this suspension by serial dilutions and plating on chocolate agar plates. The bacteria and macrophages were allowed to co-incubate for 2 h at 37°C with 5% CO_2_. Next, the medium containing the extracellular bacteria was aspirated and replaced with fresh tissue culture medium supplemented with 25 μg/ml of gentamicin for an additional 2 h. After this incubation, samples from the tissue culture wells were washed three times with PBS. The monolayer was then lysed with 200 μl of sterile water, immediately scraped, and suspended in 800 μl of PBS. The suspension was serially diluted in PBS and plated onto chocolate agar plates. The remaining tissue culture wells were assayed for CFU recovery at the 24 h post-challenge time point as described above. Replicate data from three separate experiments were normalized for comparing strains by determining the difference in percent CFU recovery between the assayed 4 and 24 h time points. Each experiment was performed in triplicate wells, wherein CFU counts were averaged from two plates per well.

### Animal Challenges

To determine the ability to cause infection, groups of 10 BALB/c mice (6–8 weeks-old and obtained from Charles River Laboratories; Frederick, MD, United States) were challenged via the intranasal route. Mice were anesthetized with approximately 150 μl of ketamine, acepromazine, and xylazine injected intraperitoneally and challenged by intranasal instillation with 50 μl of *F. novicida* suspended in PBS from 18 h grown freshly from swabbed plate cultures. Challenge doses were determined by serial dilutions in PBS and plating on chocolate agar. Mice were monitored several times each day for 21 days, and mortality rates (or euthanasia when moribund) were recorded. For re-challenge studies, mice surviving the initial *F. novicida slt* exposure (*n* = 10, and *n* = 6 for the 620 CFU group) were challenged intranasally after 28 days with the *F. novicida* parent strain or the *F. tularensis* Schu S4 strain. Survival was followed for 14 days after challenge. For dissemination experiments, infected mice were sacrificed at specific time points post-challenge, and spleens and lungs were harvested to determine bacterial CFU counts.

### Spleen Cell Preparation

Spleens were excised from mice and homogenized in CTL-medium (Cellular Technology Limited, Cleveland, OH, United States) to make the spleen extract. The spleen homogenate was diluted with CTL-medium and cells pelleted by centrifugation at 1,200 rpm for 10 min. Red blood cells in the spleen homogenate were lysed with 4 ml of ACK (Ammonium-Chloride-Potassium) Lysing Buffer (Gibco, Grand Island, NY, United States) for 5 min after which the homogenate was diluted with 10 ml CTL-medium to stop lysis. Large particulates were allowed to settle and supernatant, containing splenocytes, was poured off and centrifuged at 1,200 rpm for 10 min. The splenocytes were re-suspended in CTL-medium and counted.

### Cytokine/Chemokine Expression

Cytokines and chemokines in mouse splenocytes were measured by Luminex Mag Pix (Life Technology, Grand Island, NY, United States) as per manufacturer’s instructions. Samples were collected from BALB/c mice infected intranasally with mock/PBS (*n* = 5), 10 (*n* = 5), 94 (*n* = 5), and 935 (*n* = 3) CFU Fn *slt* mutant. Splenocytes were seeded at a concentration of 10^6^ cells/well in the presence of stimuli (medium only, irradiated *F. novicida slt*, irradiated *F. tularensis* Schu S4) in complete medium [RPMI 1640 medium containing 10% heat-inactivated fetal calf serum (Life Technology), 1 mM sodium pyruvate, 0.1 mM non-essential amino acids, 100 U/ml of penicillin, 100 μg/ml streptomycin, and 50 μM 2-mercaptoethanol] and incubated for 48 h at 37°C. Supernatants were harvested 48 h post and the levels (pg/ml) of the following cytokines/chemokines were measured in quadruplicate using ProcartaPlex Mo Cytokine/Chemokine Panel 1A 36plex (Invitrogen, 360-26092-901): IFN gamma; IL-12p70; IL-13; IL-1 beta; IL-2; IL-4; IL-5; IL-6; TNF alpha; GM-CSF; IL-18; IL-10; IL-17A; IL-22; IL-23; IL-27; IL-9; GRO alpha; IP-10; MCP-1; MCP-3; MIP-1 alpha; MIP-1 beta; MIP-2; RANTES; Eotaxin; IFN alpha; IL-15/IL-15R; IL-28; IL-31; IL-1 alpha; IL-3; G-CSF; LIF; ENA-78/CXCL5; M-CSF. We reported only on the cytokines/chemokines that showed statistically significant change relative to mock/PBS control group re-stimulation during the study.

### Enzyme-Linked Immunospot (ELISPOT)

Mouse IFN-γ enzymatic ELISPOT (Cellular Technology Limited, Cleveland, OH, United States) assay was performed by seeding purified splenocytes in the presence of stimuli as described above. Briefly, 96-well plates were coated overnight at 4°C with 80 μL/well capture anti-mouse IFN-γ monoclonal antibody. Plates were washed once with 1× PBS. Irradiated *F. novicida slt* or irradiated *F. tularensis* Schu S4 (5 μg/well) were re-suspended in CTL-medium with 1% L-Glutamine in duplicate, and 100 μl was added to each well. The plates were incubated at 37°C, 9% CO_2_ for 15 min. Splenocytes were resuspended in CTL-medium with 1% L-Glutamine and seeded at 10^4^ cells per well. Plates were incubated for 24 h at 37°C 9% CO_2_ and splenocytes were removed and plates were washed twice with PBS alone and then twice with 0.05% Tween in PBS (Tween-PBS). 80 μL/well biotinylated detection anti-mouse IFN-γ monoclonal antibody was added. After 2 h incubation at room temperature (RT), plates were washed three times with Tween-PBS. 80 μl of Strep-AP antibody solution was added to the wells, and the plates were incubated for 30 min at RT. The plates were washed twice with Tween-PBS and twice with deionized water. Development reagents were added and incubated for 15 min at RT according to manufacturer recommendations. The colorimetric reaction was stopped by washing the plates three times with distilled water and air drying overnight. Spots were scanned and analyzed using an automated ELISPOT reader (CTL-Immunospot S6 Analyzer, CTL, Germany). The T cell response was assessed as spot forming cells (SFC), adjusted to 10^6^ cells per well, which was automatically calculated by the ImmunoSpot^®^ Software for each stimulation condition and the medium only control.

### Enzyme-Linked Immunosorbent Assays (ELISA)

Sera were collected from BALB/c mice vaccinated with mock/PBS (*n* = 5), 10 (*n* = 5), 94 (*n* = 5), and 935 (*n* = 3) CFU Fn *slt* mutant. Immunoglobulin (Ig) class IgG, IgG1, and IgG2a titers in challenged mice were determined by an ELISA performed in 96-well, Immulon 2 HB, round-bottom plates (Thermo Fisher Scientific, Waltham, MA, United States), as described previously (Trevino et al., 2018). Briefly, irradiated *F. novicida slt* and *F. tularensis* Schu S4 cells used as antigens, were diluted in 0.1 M carbonate buffer (pH 9.5) to a concentration of 10 μg/ml, and 50 μl of antigens were placed into wells. Plates were stored overnight at 4°C. The plates were washed with washing solution (1× PBS, 0.05% Tween 20), and incubated with 100 μl of blocking solution (1× PBS, 1% Casein) for 30 min at 37°C. Twofold dilutions of mouse sera were made with antibody assay diluent (1× PBS, 0.25% Casein) in triplicate, and plates were incubated for 1 h at 37°C. After the plates were washed, 50 μl of 1/5,000-diluted anti-IgG-, anti-IgG1- or anti-IgG2a-horseradish peroxidase conjugate (Southern Biotechnology Associates, Inc., Birmingham, AL, United States) was added to each well, and plates were incubated for 30 min at 37°C. After the plates were washed, 50 μl of a buffered hydrogen peroxide and 3,3′,5,5′-tetramethylbenzidine solution (Pierce, Thermo Fisher) was added to each well, and plates were incubated for 20 min at 37°C. The reaction was stopped with 25 μl of 2 N sulfuric acid, and the amount of bound antibody was determined colorimetrically by reading absorbance at 450 nm with a reference filter (570 nm). The results are reported as the reciprocal of the highest dilution giving a mean OD of at least 0.1 (which was at least twice the background) ± 1 SD.

### Statistics

For comparing data from the sensitivity to inhibitor and CFU recovery from macrophages, statistical significance (*p* < 0.05) was determined by the two-tailed Student *t*-test. Growth analysis of bacterial strains in broth media was analyzed as previously described (Zwietering et al., 1990). A logistic growth equation was used to fit the data as a function of maximum density, and maximum growth rate. The resulting estimates were entered into a two-way ANOVA to analyze the effect of pH and mutation. CFU counts were compared between strains by one-way ANOVA, applied to the log transformed data. For mouse challenge experiments, LD_50_ values were determined by the Bayesian probit analysis, and association between median time to death and dose is modeled by log-logistic parametric survival regression models. For immune studies, comparison to control groups (PBS only) was made by *t*-test applied to log transformed values.

## Results

To determine the role of the peptidoglycan-modifying Slt enzyme in virulence of *Francisella*, we initially focused on mutagenesis of the *slt* gene in LVS and the fully virulent *F. tularensis* Schu S4 strains. Targetron-based mutagenesis was attempted using two different plasmids targeting interruption of the gene in positions 267 and 418 of the ORF. Colonies were obtained only for the plasmid targeting position 418, and PCR screening revealed the presence of both WT and intron-containing mutant alleles. However, after passaging and subsequent screening of at least 50 colonies, a pure mutant was not able to be isolated (data not shown). Similarly, a pMP590-based suicide plasmid containing an in-frame deletion of the *slt* gene with flanking homologous sequences, failed to produce mutant colonies. At least 12 co-integrates containing both WT and mutant alleles were obtained from the initial electroporation, but subsequent passaging on sucrose and screening of approximately 250 colonies for the second recombination event yielded only WT revertants (data not shown). These results suggested *slt* is an essential gene in LVS and *F. tularensis*. However, from a transposon mutant library previously described in *F. novicida*, a T20 transposon insertion in the *slt* gene occurred at position 479 (Gallagher et al., 2007). Therefore, we focused on the *slt* gene in *F. novicida*, a commonly used surrogate for *F. tularensis*.

**FIGURE 1:**
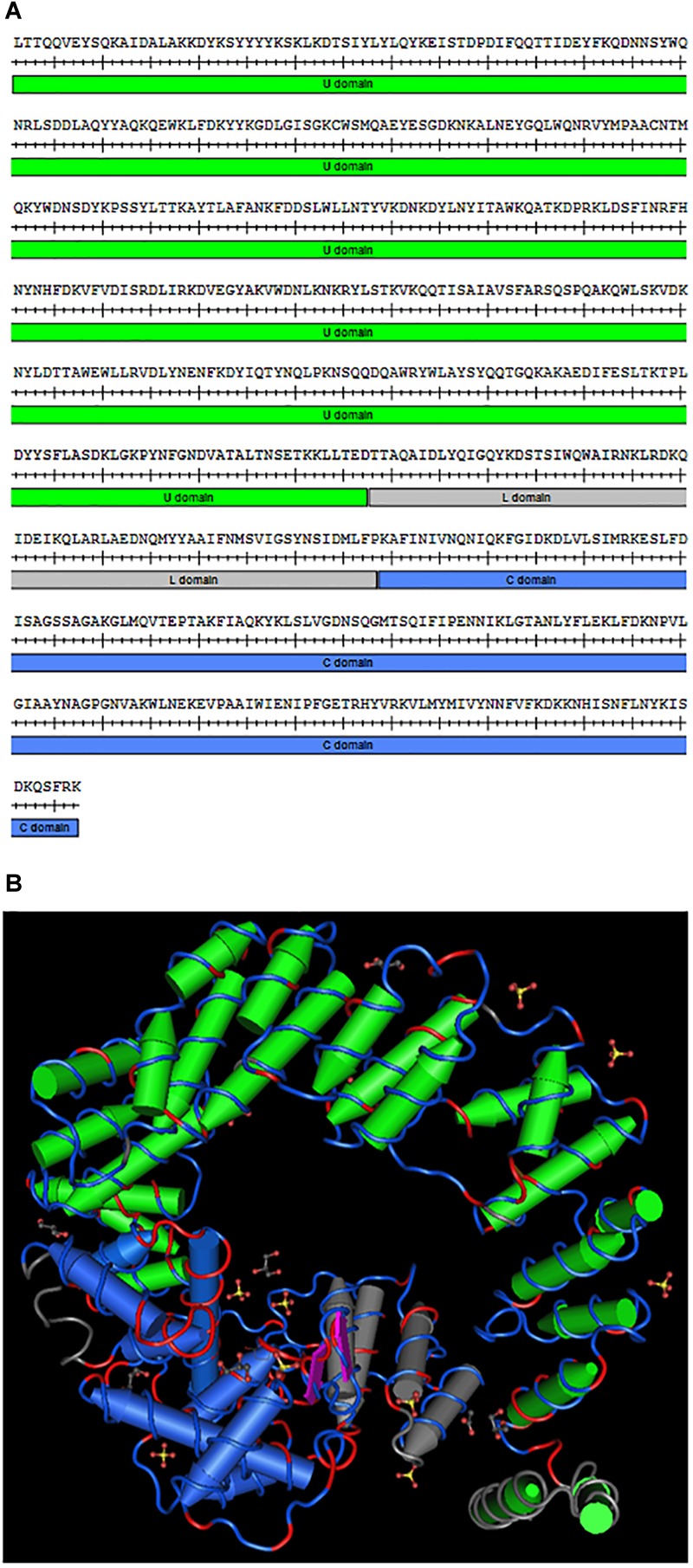
*Francisella novicida* Slt homology model. **(A)** Amino acid sequence of the mature *F. novicida* Slt protein; U domain, linker (L) domain, and catalytic (C) domain are annotated in green, gray, and blue, respectively. **(B)** Homology model of *F. novicida* Slt onto the structure of Slt70 from *E. coli*. U, L, and C domains are color-coded according to sequence shown in **(A)**. Model was generated using Cn3D software v4.3.1 based on alignment of *F. novicida* Slt with the crystal structure of Slt70 from *E. coli* (PDB accession 1QSA). The helix cylinders are color-coded according to the domains shown in **(A)**. The amino acid backbone is rendered according to identity; identical residues in red and dissimilar residues in blue.

The *slt* gene in *F. novicida*, encoding the soluble lytic transglycosylase, is 1,977 bp long and spans the positions 501311–503287 on the chromosome (FTN_0496). The *slt* gene lies upstream of FTN_0497, encoding a hypothetical protein, and downstream of FTN_0495, encoding a BNR (bacterial neuraminidase repeat) repeat-like domain protein, both located on the opposite strand. Therefore, *slt* is unlikely to be part of an operon. A BLASTp search revealed the Slt protein shares 24% sequence identity with the murein transglycosylase Slt70 from *E. coli* and is part of the MltE superfamily (data not shown). Slt70 has been shown to function as an exo-muramidase, releasing disaccharides from the glucosamine end of peptidoglycan fragments (Romeis et al., 1993). The amino acid sequence of *F. novicida* Slt is shown in Figure 1A, with the U-shaped U domain, linker (L) domain, and catalytic (C) domain annotated. A homology model of the protein was generated based upon the crystal structure of Slt70 from *E. coli* (van Asselt et al., 1999) (Figure 1B). Conserved residues are shown in red on the amino acid backbone; despite low sequence identity, the proteins exhibited a similar structure, primarily dominated by alpha-helices. The structural similarity suggests the *F. novicida* Slt enzyme may have similar function to that of *E. coli*.

We obtained the *F. novicida slt* mutant from the aforementioned transposon library, and complemented the mutant *in-trans* using the pMP822 vector containing the parent *slt* allele. These strains were then utilized to investigate the impact of *slt* inactivation on *Francisella* growth, cell biology, and virulence *in vivo*. Moreover, we assessed the immune response against the *slt* mutant and determined its efficacy as a vaccine candidate to protect against challenge with either the parent *F. novicida* or fully virulent *F. tularensis* Schu S4 strain.

### Inactivation of *slt* Results in Significant Growth Defects in Low pH Conditions

**FIGURE 2:**
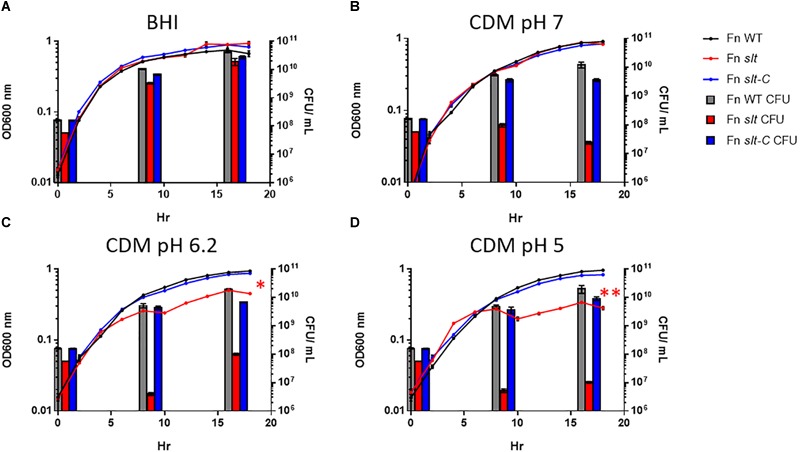
*Francisella novicida slt* exhibits pH-dependent growth defects. *F. novicida* WT, *slt*, and *slt*-C were grown in BHI broth **(A)** and Chamberlain’s defined medium adjusted to pH 7 **(B)**, 6.2 **(C)**, or 5 **(D)**, and monitored by both optical density and plating for CFU at specific time points. OD_600nm_ readings are shown as points with connecting lines, while CFU/mL at 0, 8, and 16 h growth are shown as bars. Results represent average with standard error of the mean based on quadruplicate well measurements. These data are representative of at least three experiments. CFU counts were compared using one-way ANOVA applied to log-transformed data; counts for the *slt* strain were significantly different from WT at 0, 8, and 16 h in all conditions tested (red bars). OD values were fitted to a logistic growth equation, and fold-change was determined by two-way ANOVA; ^∗^*P* ≤ 0.01, *slt* compared to WT and *slt*-C, ^∗∗^*P* ≤ 0.001, *slt* compared to WT and *slt*-C.

In order to determine the impact of the *slt* gene on cell growth, we performed growth curves comparing *F. novicida* WT (Fn WT), *slt* mutant (Fn *slt*), and complemented (Fn *slt*-C) strains in both BHI broth and CDM. Our initial experiments revealed a decrease in growth of the *slt* mutant in CDM, as measured by optical density, while growth in BHI was similar to WT (Figure 2A,C). Given the pH of standard CDM at 6.30 ± 0.1 versus BHI broth at pH 7.4 ± 0.2, we hypothesized the *slt* mutant may have pH-dependent differences in growth (Chamberlain, 1965; Shanson and Singh, 1981). Therefore, growth of Fn WT, *slt*, and *slt-*C strains were compared in CDM adjusted to pH 5.0, 6.2, and 7.0 and BHI broth. Optical density (OD) readings across all three strains were similar in both BHI broth and CDM pH 7.0 (Figure 2A,B). Interestingly, the *slt* mutant consistently displayed ∼2–3-fold fewer CFU counts than WT or complement strain when adjusted to the same OD (Figure 2, bars at 0 h). However, in BHI broth, the CFU counts for the mutant increased and were not significantly different as compared to WT at points tested later in the experiment. During growth in CDM adjusted to pH 7.0, we observed no increase in CFU counts for Fn *slt*, even though growth kinetics as assessed by OD mirrored WT and complement strains. In CDM pH 5.0 and 6.2, the *slt* mutant was significantly affected in growth by both OD and CFU measurements, while the WT and complement strains had similar growth kinetics in all pH conditions (Figure 2C,D). These results indicate the *slt* mutant is significantly impaired for growth in CDM broth, especially at lower pH conditions.

### Scanning Electron Microscopy Analysis of *F. novicida slt* Mutant Reveals Altered Cell Morphology at Low pH

**FIGURE 3:**
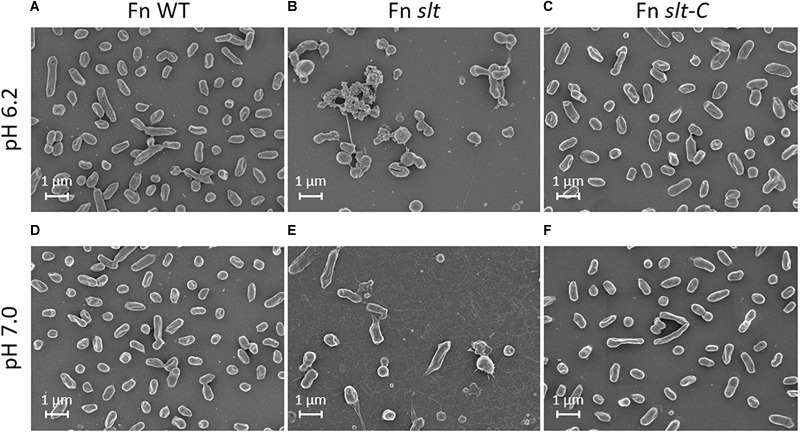
Scanning electron microscopy analysis of Fn strains. Fn WT, *slt*, and *slt*-C strains were grown to exponential phase in CDM adjusted to pH 6.2 (top panels; **A–C**), or pH 7.0 (bottom panels; **D–F**), and analyzed by SEM. Images were acquired under 10,000× magnification; scale bar, 1 μm.

**FIGURE 4:**
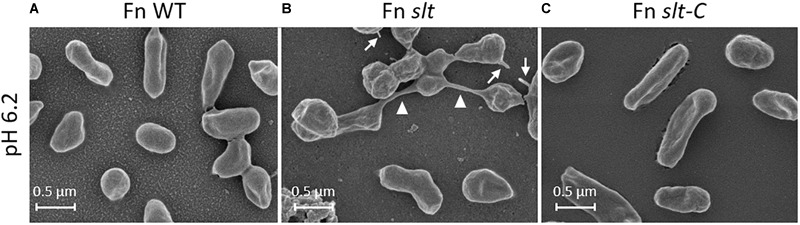
Surface structure and morphology of Fn strains. Fn *slt* cells grown in CDM pH 6.2 show significantly ruffled surface structure and fusion of cells (**B**, arrowheads), as well as tube-like surface projections (**B**, arrows). Equal magnification images of Fn WT **(A)** and Fn *slt*-C **(C)** strains grown in CDM pH 6.2 show smooth surface appearance of cells and uniform appearance. Images were acquired under 30,000× magnification; scale bar, 0.5 μm.

In order to determine how loss of the *slt* gene affects cellular structure, SEM was performed on Fn WT, *slt*, and *slt*-C strains grown in CDM pH 6.2 or 7. Wild-type cells grown at pH 6.2 and or 7 displayed typical coccobacilli appearance, with a mixture of bacilli and cocci with a smooth surface appearance (Figure 3A,D). At pH 6.2, Fn *slt* displayed dramatically altered cell morphology including aberrant cell shapes and sizes, rough surface appearance, and extensive clumping of cells (Figure 3B). In contrast to WT, *slt* cells were often larger and more spherical, and exhibited tube-like surface protrusions (Figure 4B, arrows). Additional high magnification images of the *slt* mutant at pH 6.2 also show extensive tube-like membrane projections (Figure 4B, arrows) and tube-like connections between cells (Figure 4B, arrowheads), which may represent outer membrane tubes. Significant ruffling of the *slt* mutant cell surface is also apparent in the high magnification images, in contrast to the smooth appearance of Fn WT cells grown in the same conditions (Figure 4). Interestingly, growth of Fn *slt* at pH 7 partially restored these defects, resulting in more rod-shaped cells with smoother surface appearance, although cocci with surface protrusions were still evident (Figure 3E). Importantly, the *slt*-C strain was appreciably similar to WT in both pH conditions, indicating complementation had restored cell morphology (Figure 3C,F). However, a small portion of the *slt*-C population had much longer rod-shaped bacteria than WT.

Additionally, extensive fibrous material was observed in the background of *slt* mutant pH 7 images, and to a lesser extent in pH 6.2 images, while absent in the other strains. The significance of this material is unknown, but is not likely to be an artifact of sample preparation since all samples were processed at the same time. Overall, these SEM studies uncover a critical role for Slt in maintaining proper cell shape and cell division in low pH conditions.

### *F. novicida slt* Exhibits Reduced Cell Viability During Growth in Low pH Conditions

**FIGURE 5:**
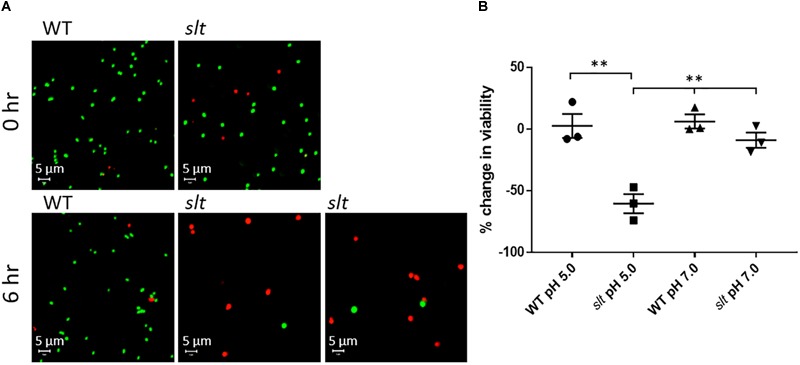
Live/Dead staining analysis of Fn WT and *slt*. Cells were grown in CDM adjusted to either pH 5.0 or 7.0 and subjected to Live/Dead staining and confocal microscopy at 0 and 6 h time points. **(A)** Representative confocal images of WT and *slt* strains grown in CDM pH 5.0. **(B)** The percentage of viable cells was calculated as number of green-stained cells divided by the total number of cells, and then used to determine the percent change in viability from 0 to 6 h of growth. The mean and standard error were plotted for three independent experiments, wherein at least 100 cells were counted for each sample. Significance was determined by student’s *t*-test; ^∗∗^*P* < 0.01 for *slt* pH 5.0 compared to each other sample.

As shown above, the *slt* mutant demonstrated decreased CFU recovery during growth in CDM. Two potential reasons for this are decreased cell viability or clumping of the cells. SEM analysis showed the *slt* mutant had increased clumping. To assess whether viability was also affected, we utilized LIVE/Dead staining on Fn strains grown in CDM adjusted to pH 5.0 or 7.0. Representative confocal images and percent change in viability from 0 to 6 h for each sample are shown in Figure 5. Viability of the *slt* mutant in CDM pH 5.0 exhibited a significant decrease of 60%, while the viabilities for Fn WT in both pH conditions increased slightly (2% in pH 5, 6% in pH 7).

At neutral pH, the *slt* mutant showed a slight decrease of viability, which was not significantly different from WT. Additionally, on average, the initial viability of the *slt* mutant was slightly lower than WT, ∼80% compared to ∼90%, which may elucidate why initial CFU counts for the *slt* mutant ran slightly lower than WT at equivalent OD values. Additionally, and in agreement with SEM results, we observed increased cell sizes of the *slt* mutant at 0 h compared to WT, which continued to expand at 6 h (Figure 5A). This likely explains the reduced CFU counts of the *slt* mutant in previous growth curves, despite the increase in OD values.

### The *F. novicida slt* Mutant Exhibits Altered Antimicrobial Sensitivities

In order to determine the impact of *slt* inactivation on susceptibility to antimicrobials and membrane integrity, the MIC of polymyxin B was assessed using *E*-tests. Interestingly, the *slt* mutant displayed an increased resistance to polymyxin B, with an MIC of >1024 mg/L in contrast to 96 mg/L for WT. In this assay, the *slt*-C strain also showed an increased MIC of >1024 mg/L, indicating this phenotype could not be reversed by complementation. The MIC values are based upon 3 independent assays performed in triplicate wells. Sensitivity to membrane inhibitors Tween20, Triton X-100, sodium dodecyl sulfate, and sodium deoxycholate was also tested, but no appreciable difference between WT and *slt* mutant was observed (data not shown).

### The *slt* Mutant Exhibits Impaired Intracellular Replication in Macrophages

**FIGURE 6:**
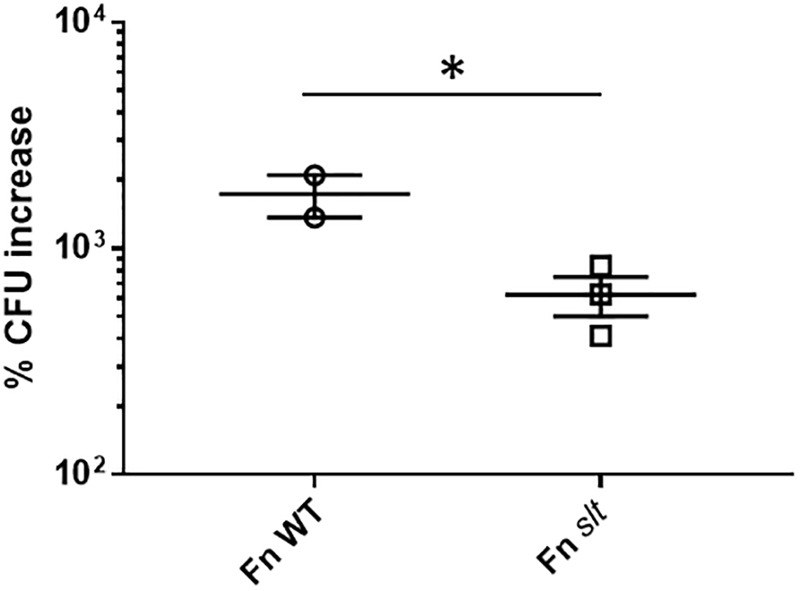
Replication of *F. novicida* in J774.1 murine macrophages. Macrophages were infected with WT or *slt* strains at an MOI of ∼100:1 and incubated in the presence of gentamicin to eliminate extracellular bacteria. At 4 and 24 h time points, cells were lysed and plated for recovered bacteria, and percent increase of bacteria from 4 to 24 h was calculated. Results from three independent experiments performed in triplicate wells are shown, and statistical significance was determined by student’s *t*-test; ^∗^*P* < 0.05.

Given the impact of *slt* inactivation on cell growth and division of Fn in acidic conditions, we wanted to determine whether growth in macrophages, where bacteria are exposed to acidic environments, would also be affected. J774.1 murine macrophages were infected with Fn WT or *slt* for 4 to 24 h and treated with gentamicin to eliminate extracellular bacteria. Macrophages were then lysed and plated to enumerate intracellular bacteria. Data are presented as percent CFU increase, indicating the differences in CFU recovery between 4 and 24 h for each strain. A ∼625% CFU increase was observed for the *slt* mutant inside macrophages, showing the mutant still retained the ability to replicate intracellularly. However, the level of replication was significantly decreased, 2.8-fold lower, than that of the parent strain (Figure 6). These results show that the Slt enzyme contributes to the ability of *F. novicida* to replicate intracellularly within macrophages, and therefore could be affected for virulence during infection of the host.

### The *slt* Mutant Is Highly Attenuated in Mice

**FIGURE 7:**
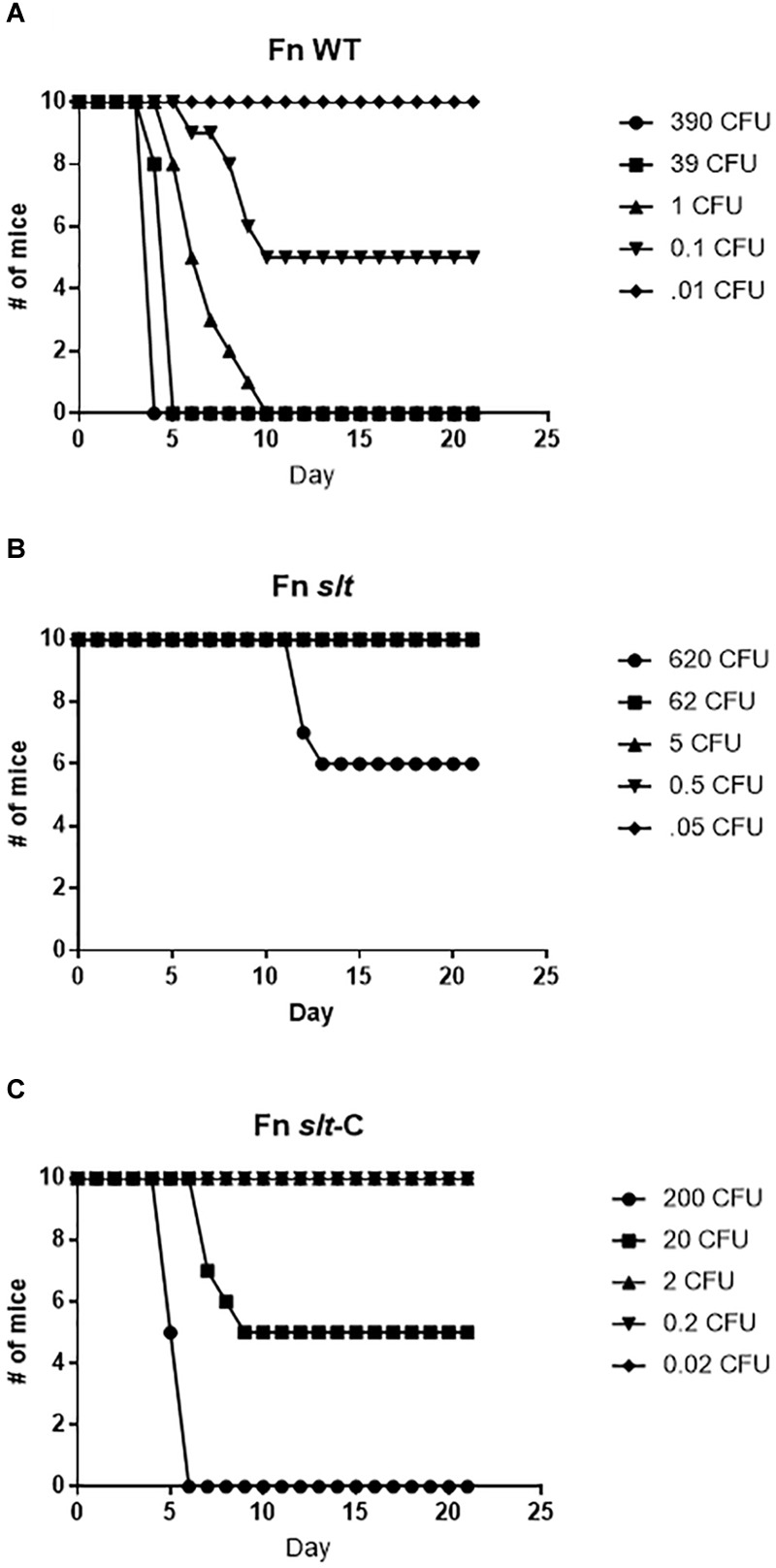
Fn *slt* is attenuated during intranasal infection. Groups of BALB/c mice (*n* = 10) were challenged with 5 doses of **(A)** Fn WT, **(B)** Fn *slt*, and **(C)** Fn *slt*-C strains, and monitored for 21 days. LD_50_ values from these experiments are included in Table 2.

To assess the contribution of the *slt* gene to *Francisella* virulence, BALB/c mice were challenged via the intranasal route with various doses of either the WT strain or the *slt* mutant. As previously demonstrated, BALB/c mice were highly susceptible to challenge with the parent strain, and the LD_50_ was calculated to be less than 1 CFU (Figure 7 and Table 2) (Chance et al., 2017). In contrast, the *slt* mutant was shown to be highly attenuated in the murine model, and a LD_50_ value was not able to be measured as only 4/10 mice succumbed to infection at the highest challenge dose (620 CFU). In addition, for those mice that did succumb to infection with the *slt* mutant (*n* = 4), the time to death was significantly greater than mice which succumbed to challenge with the parent (*n* = 10) at approximately similar doses (∼400 CFU), 12.5 days versus 4.25 days, respectively. Importantly, complementation of the mutant nearly restored virulence, as shown by the LD_50_ of 20 CFU, indicating the attenuation was due specifically to the loss of the *slt* gene (Figure 7).

**Table 2:** Lethal dose calculations for intranasal infection with *F. novicida* strains.

Strain	LD_50_	*P*-value
U112	<1 CFU	
*slt*	>620 CFU	<0.0001^∗^
*slt*-C	20 CFU	<0.0001^∗∗^

*^∗^P-values are based on pairwise comparisons made under a probit model, assuming a common slope of the dose-response curve over the course of 21 days. ^∗^P-value comparing LD_50_ values between WT and slt mutant. ^∗∗^P-value comparing LD_*50*_ values between slt mutant and complemented strain*.

### The *F. novicida slt* Mutant Exhibits Significantly Reduced Dissemination in a Murine Model of Intranasal Infection

**FIGURE 8:**
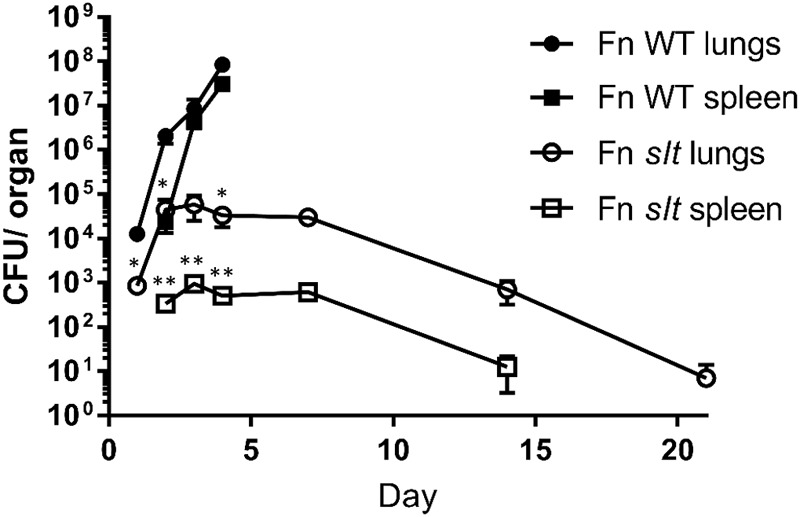
Dissemination of *F. novicida* WT and *slt* strains during intranasal challenge. BALB/c mice were infected intranasally with 42 CFU WT or 52 CFU *slt* mutant. At days 1, 2, 3, 4, 7, 14, and 21, mice were euthanized and lungs and spleens were harvested. Serial dilution and plating of homogenized lung and spleen were performed to determine recovery of bacteria. Results are shown as CFU per organ based on 5 mice per time point, except for day 4 WT; only four Fn WT-challenged mice remained and the rest had succumbed to infection. Error bars represent standard error of the mean; ^∗∗^*P* < 0.001, average CFU/spleen days 2, 3, 4; ^∗^*P* < 0.05 average CFU/lung days 1, 2, 4, as assessed by *t*-test. WT-infected mice were only carried out until day 4, based on previous experiments showing a median time until death of 5 days for WT *F. novicida*.

To determine the ability of the mutant strain to traffic from the lung and then replicate within organs of the mice following challenge, we performed dissemination studies to compare the bacterial burden of WT versus *slt* mutant strains. BALB/c mice were infected via the intranasal route with 42 CFU of WT or 52 CFU of Fn *slt*, and euthanized for sample collections as indicated (Figure 8). Bacterial burden in the lungs of WT-infected mice increased from ∼1.3 × 10^4^ CFU on day 1 to ∼8.4 × 10^7^ CFU on day 4 (Figure 8). After day 4, no WT-infected mice were available as all had succumbed to infection. By day 2, bacteria were detected in the spleen of WT-infected mice, and CFU values increased from ∼2.4 × 10^4^ to ∼3.1 × 10^7^ on day 4. In contrast, mice receiving the *slt* mutant displayed minor or no clinical signs, and all survived challenge out to day 21. Furthermore, bacterial burden in the lung of *slt*-infected mice only increased slightly from ∼8.5 × 10^2^ on day 1 to ∼5.8 × 10^4^ CFU on day 3, and then steadily declined the following days until only few colonies could be recovered on day 21 (Figure 8). Bacterial burden in the spleens of *slt*-infected mice followed similar kinetics, with no bacteria being recovered at day 21. Since the bacterial burden in the lungs on day 1 was significantly lower in *slt*-infected mice than in WT-infected mice, despite equal challenge doses, these data suggest impaired bacterial replication and/or survival in the lungs. Therefore, we debated if this apparent attenuation of the *slt* mutant strain could allow for its use as a live vaccine and possibly offer protection from challenge with the parent Fn strain. If successful, we would then proceed to assess protection against a fully virulent *F. tularensis* strain.

### The *F. novicida slt* Mutant Protects Against WT *F. novicida* Challenge

**FIGURE 9:**
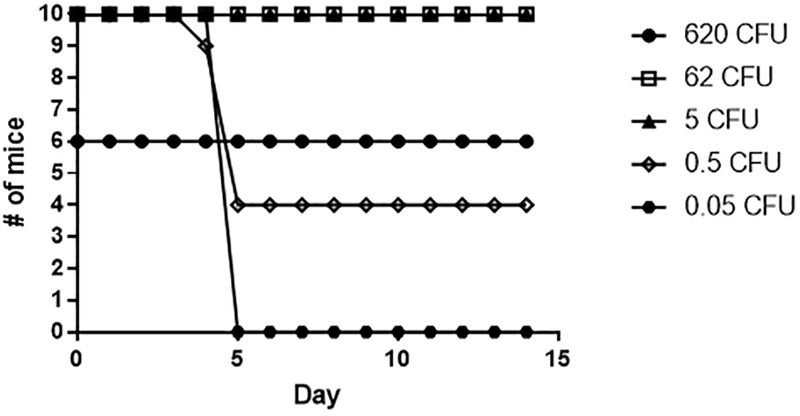
Fn *slt* provides protection against challenge with Fn WT. Mice surviving the initial Fn *slt* exposure (*n* = 10, and *n* = 6 for the 620 CFU group) were challenged after 28 days with 35 CFU of the Fn parent strain.

To determine if the *slt* mutant would provide protection against challenge with the Fn parent strain, mice which survived exposure to the *slt* mutant were challenged after 28 days with the Fn parent strain via the intranasal route (Figure 9). Mice that received 5 CFU or greater of the Fn *slt* mutant were completely protected against WT challenge. Only groups that received on average less than 1 CFU of Fn *slt* had mice that succumbed to infection. The 50% protective dose (PD_50_) of Fn *slt* was determined to be 0.537 CFU by probit regression analysis, and significant protection relative to mice challenged with 35 CFU of the parent Fn strain was observed at Fn *slt* doses of 0.2 CFU or greater (*P* ≤ 0.0001). It is estimated that mice receiving the *slt* mutant and challenged with the parent strain had a median time to death 3.38 times greater than naïve mice challenged with the parent strain. These results highlight the potential of a *slt* mutant strain to protect against infection with *Francisella*.

### The *F. novicida slt* Mutant Induces a Stronger Th17- and Th2-Type Recall Immune Response Relative to Th1-Type Response in the Murine Model of Intranasal Infection

**FIGURE 10:**
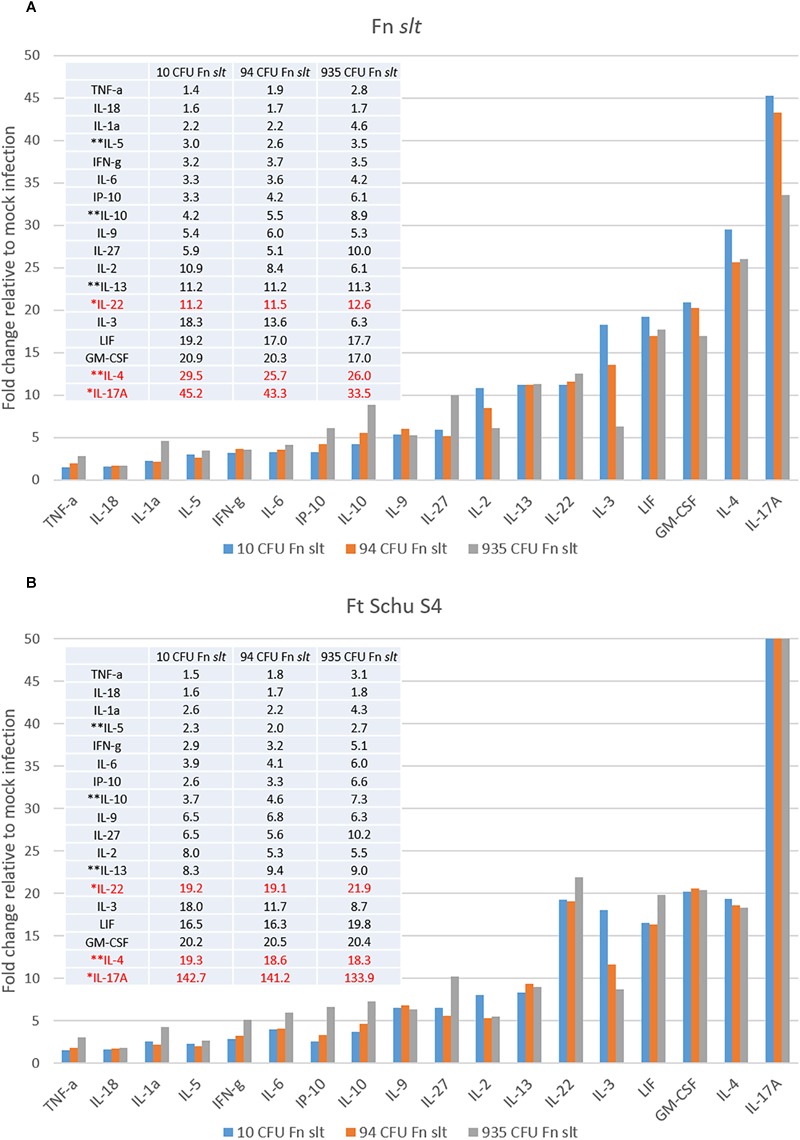
Robust Th17- and Th2-type cytokine response in splenocytes re-stimulated with Fn *slt* or Ft Schu S4. BALB/c mice were infected intranasally with mock/PBS (*n* = 5), 10 (*n* = 5), 94 (*n* = 5), and 935 (*n* = 3) CFU Fn *slt* mutant. At day 21 post infection, mice were euthanized and spleens were harvested. Splenocytes (10^6^ cells) were re-stimulated for 48 h in the presence of **(A)** irradiated Fn *slt* (5 μg/ml) or **(B)** Ft Schu S4 (5 μg/ml) and the levels of cytokines measured by the Luminex bead-based suspension assay. Graph and Table values are expressed as a fold change compared to PBS control group for each analyte. Red text indicates cytokine profiles which markedly differed between Fn *slt* and Ft Schu S4 re-stimulation. ^∗^Th17-like cytokines; ^∗∗^Th2-like cytokines. A *P*-value of <0.05 was determined for every analyte by *t*-test of log10 values against PBS only controls.

In an effort to identify immune correlates of protection and evaluate the protective efficacy of the Fn *slt* mutant as a surrogate vaccine against a highly virulent Tier 1 strain, mice were vaccinated with Fn *slt* mutant and challenged with *F. tularensis* Schu S4. Groups of BALB/c mice (*n* = 20) received 10, 94, or 935 CFU of Fn *slt* strain by the intranasal route. Another group of 20 mice received PBS alone as naïve controls. At day 21 post infection, 3 or 5 mice from each group were euthanized and spleens were harvested. To assess the recall immune response, splenocytes were re-stimulated in the presence of irradiated Fn *slt* or *F. tularensis* Schu S4 (Ft Schu S4). Of the analytes tested, the secretion levels of 18/36 cytokines were significantly increased, relative to mock (PBS) vaccinated control group under both Fn *slt* and Ft Schu S4 re-stimulation conditions (Supplementary Figure S1). The results are expressed as fold change relative to the mock (PBS) vaccinated control group for all three vaccine doses. Upon re-stimulation with Fn *slt*, there was a strong induction of IL-17A, IL-4, GM-CSP, LIF, IL-3, IL-22, and IL13 (Figure 10A). Re-stimulation with Ft Schu S4 produced a similar overall cytokine profile compared to Fn *slt* with the exception of IL-17A, IL-4, and IL-22 (Figure 10B). Of all the cytokines tested, the largest fold increase under both re-stimulation conditions was with IL-17A. However, it was much more elevated upon re-stimulation with Ft Schu S4 compared to Fn *slt* (133.9–142.7- vs. 33.5–45.3-fold). The change in Th17-like cytokines (IL-17A and IL-22) was prominent with both Fn *slt* and Ft Schu S4 re-stimulation. While IL-4 secretion increased in splenocytes re-stimulated with Fn *slt* compared to Ft Schu S4 (25.7–29.5- vs. 18.3–19.3-fold), the change in secretion of IL-22 was less pronounced (11.2–12.6- vs. 19.1–21.9-fold). Furthermore, the overall changes in Th2-like cytokine (IL-4, IL-5, IL-10, and IL-13) secretion was greater than Th1-like cytokines (TNF-α, IFN-γ, and IL-2) under both re-stimulation conditions. Re-stimulation with Fn *slt* also produced a dose-dependent enhancement in TNF-a, IL-1a, IP-10, IL-10, and IL-27 cytokine secretion and a dose-dependent reduction in IL-2, IL-3, IL-4, and IL-17a secretion. Similar trends are also evident with Ft Schu S4 re-stimulation and additional enhancements in IFN-γ, IL-6, IL-22, and LIF secretion are observed at the highest dose of Fn *slt* vaccine.

We observed a 3.2–3.7-fold increase in IFN-γ secretion for the three Fn *slt* vaccine groups in comparison to the PBS mock-infected control group. Furthermore, the concentration of IFN-γ for the three Fn *slt* vaccine groups was markedly elevated (1624.9–1866.0 Fn *slt* vs. 504.4 PBS). This pronounced IFN-γ response was confirmed with a high number of IFN-γ producing T-cells (*p* < 0.0001 vs. PBS) under both re-stimulation conditions in an ELISpot assay, although there was no difference in the number of IFN-γ secreting cells between Fn *slt* vs Ft Schu S4 re-stimulations (*p* = 0.4259 Fn *slt* vs. Ft Schu S4) (Supplementary Figure S2).

### The *F. novicida slt* Mutant Induces Biased Production of IgG2a Antibodies Relative to IgG1

**FIGURE 11:**
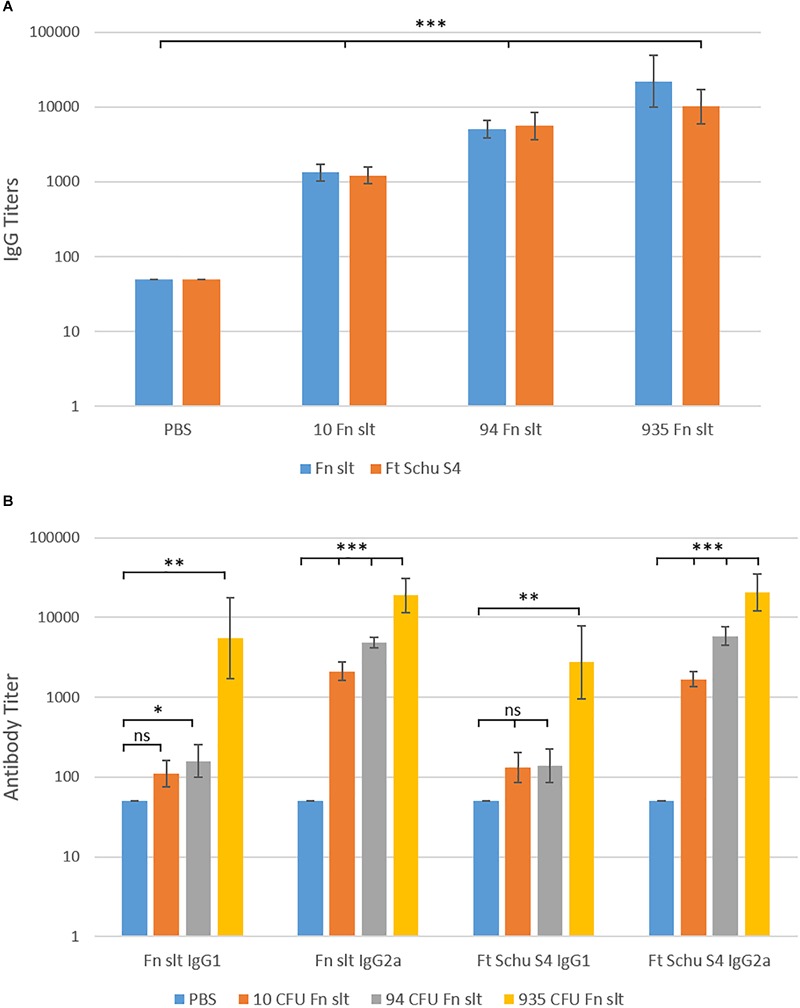
Serum IgG, IgG1, and IgG2a antibody titer levels by ELISA. Sera were collected from BALB/c mice vaccinated with mock/PBS (*n* = 5), 10 (*n* = 5), 94 (*n* = 5), and 935 (*n* = 3) CFU Fn *slt* mutant. The reactivity of **(A)** total IgG, **(B)** IgG1 and IgG2a in serum was tested against Fn *slt* or Ft Schu S4 by endpoint ELISA. Statistical significance was determined by *t*-test on log10 values compared against PBS only control; ns, not significant, ^∗^*P* < 0.5, ^∗∗^*P* < 0.01, ^∗∗∗^*P* < 0.001.

In addition to evaluating the cytokine response, sera from Fn *slt* vaccinated mice were collected in order to evaluate the antibody response generated against the irradiated Fn *slt* and Ft Schu S4 strains. An Fn *slt* vaccine mediated dose dependent increase in IgG titers was observed against both bacterial strains and the total mean IgG response peaked (Fn *slt* mean titer: 1:22,945 and Ft Schu S4 mean titer: 1:10,159) at the highest vaccine dose (935 CFU of Fn *slt*) (Figure 11A). Antibody titers for both IgG1 and IgG2a increased with increasing doses of Fn *slt* vaccine (Figure 11B). Interestingly, the IgG2a isotype response was considerably greater than IgG1 against both Fn *slt* and Ft Schu S4. This IgG2a/IgG1 difference was most apparent in mice that were vaccinated with two lower doses of Fn *slt* (10 and 93 CFU). Generally, all antibody titers were relatively low, although an IgG2a biased response was observed relative to IgG1.

**FIGURE 12:**
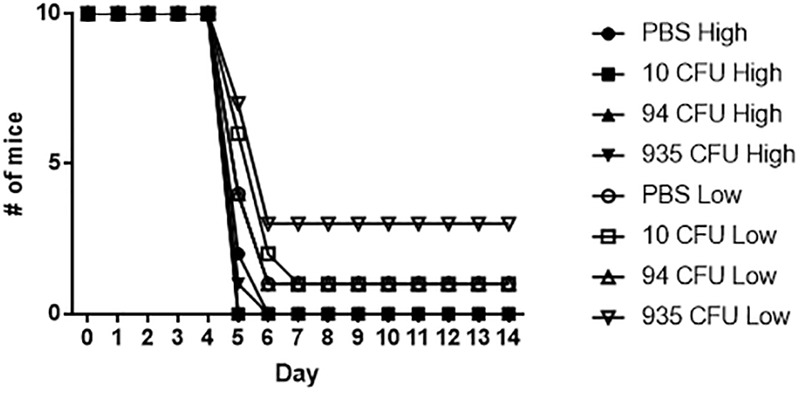
Fn *slt* does not protect against re-challenge with Ft Schu S4. Groups of mice receiving 10, 94, and 935 CFU of Fn *slt* vaccine or PBS only (*n* = 10) were challenged 28 days post-vaccination with either a high dose (37 CFU) or low dose (6 CFU) of Ft Schu S4 via the intranasal route. Log rank comparison and survival regression analyses identified no significant difference between vaccinated and control groups.

Based on the immune data collected from the vaccinated mice, we sought to determine if the mice receiving the Fn *slt* mutant would be protected against challenge with the Ft Schu S4 strain. 28 days post-vaccination, the three groups of Fn *slt* vaccinated mice and naïve control mice (*n* = 20) were split into two groups of 10 and challenged intranasally with either a high (37 CFU) or low (6 CFU) dose of Ft Schu S4. Based upon our previous published data, the IN LD_50_ for BALB/c mice is 1 CFU of Ft Schu S4 (Chance et al., 2017). However, despite the promising results with protection against Fn challenge, no protection was afforded to the mice receiving any dose of the *slt* mutant strain when challenged with the Ft Schu S4 strain (Figure 12).

## Discussion

### Role of Slt in Cell Division and Morphology

Our studies show that *slt*, encoding a soluble lytic transglycosylase, is required for proper growth and cell division of *F. novicida* in low pH conditions and, more importantly, virulence in mice. When attempting to inactivate the *slt* gene from LVS or Ft Schu S4, FTL_0466 and FTT_0400, respectively, we were unable to obtain a *slt* mutant in these background strains of *Francisella.* These results suggested that the *slt* gene was essential in these background strains. In agreement with our observation, the *slt* gene was found to be among the 453 genes essential for *in vitro* growth of Ft Schu S4 in a recent study (Ireland et al., 2019). Furthermore, a second laboratory was unable to isolate a Ft Schu S4 *slt* mutant from a transposon mutant library (personal communication, Thomas Lamkin, Wright Patterson Air Force Base). Currently, we are unsure why the *slt* gene can be inactivated in a *F. novicida* background but not in LVS or Ft Schu S4. It is possible that *F. novicida* contains additional enzymes with transglycosylase activity that partially compensate for the loss of Slt, preventing a lethal phenotype. For instance, Rohmer et al. (2007) previously reported that 84 genes encoded in the Fn genome are inactivated in both Ft Schu S4 and LVS.

Interestingly, morphological defects were observed during growth of the Fn *slt* mutant under acidic conditions. Scanning electron micrographs of the *slt* mutant at pH 6.2 revealed cells that were larger in size and appeared to have extensive clumping and fusion of cells as compared to the parent strain, which likely indicates an inability of daughter cells to separate during cell division. Supporting this concept, lytic transglycosylases in *E. coli* have been shown to contribute to cell separation by splitting the murein septum (Heidrich et al., 2002). We presume the clumping and enlarged cell sizes of the mutant may account for the decreased OD readings observed during the growth at low pH values. Since the Slt enzyme is important for peptidoglycan recycling, it is likely that buildup of peptidoglycan fragments in the outer membrane layer of *slt* mutant cells affects cellular metabolism as well as morphology. For instance, previous work on peptidoglycan recycling in *Neisseria gonorrhoeae* showed that mutation of *ampD*, involved in peptidoglycan degradation, caused a buildup of peptidoglycan monomers in the cytoplasm as well as increased metabolism of disaccharides (Garcia and Dillard, 2008).

Significant redundancy of LT enzymes is observed in many bacteria; for example, *E. coli* harbors at least six membrane-bound LTs and one soluble LT, Slt70, and *Pseudomonas aeruginosa* produces at least seven LTs (Johnson et al., 2013). It has been shown that deletion of multiple LT enzymes in *E. coli*, including the major transglycosylase Slt70, does not significantly impact morphology or growth (Heidrich et al., 2002). Interestingly, only one other murein transglycosylase was found to be annotated in the *F. novicida* genome (FTN_1286), whose amino acid sequence is highly different from that of Slt, but homologous to MltA from *E. coli* (34% identity, and 47% similarity based on BLASTp search). In contrast to *E. coli*, a study on the lytic transglycosylase LtgC of *N. gonorrhoeae*, another homolog of MltA, showed that mutation of this single enzyme significantly impacted cell growth, septation, and division (Cloud and Dillard, 2004). Moreover, the authors noted decreased CFU counts and cell viability of the mutant that were restored by complementation, similar to our results. Other cell wall-modifying enzymes have been described in *Francisella* spp. For example, Ireland et al. (2019) recently reported *ampG* and *ampD*, encoding a muropeptide transporter and a murein amidase, both involved in cell wall recycling, are essential in Ft Schu S4. Interestingly, FTT_0924, encoding an unknown protein highly conserved among *Francisella* spp., was shown to be necessary to maintaining peptidoglycan stability and for intracellular replication in macrophages (Brunton et al., 2015). Similarly, the D-alanyl-D-alanine carboxypeptidase DacD was found to be necessary for maintaining the integrity of the cell wall as well as being involved in intracellular replication and virulence in *F. holarctica* (Spidlova et al., 2018). While knowledge of the specific peptidoglycan remodeling proteins in *Francisella* is currently limited, we demonstrate that the Slt enzyme described in this study has a critical role in maintaining cell growth and morphology in acidic conditions.

The pH-dependent phenotype of the *slt* mutant is a unique finding. Recent studies on the D-alanyl-D-alanine carboxy-peptidase DacD, another peptidoglycan-remodeling enzyme, showed a significant pH sensitivity of a *F. holarctica dacD* mutant, as well as morphological defects including increased cell size up to ten times larger than WT, and discontinuous membranes that left cells more susceptible to osmotic stress (Spidlova et al., 2018). The Fn *slt* mutant cells generally appeared to be larger than WT but not to this degree as observed for the *F. holarctica dacD* mutant. The authors also demonstrate that while intracellular replication of the *dacD* mutant was affected during infection of murine macrophages, phagosomal escape was increased compared to WT, possibly due to the increased sensitivity to acidic environment of the phagosome (Spidlova et al., 2018). While our studies did not address phagosomal escape, we show that the *slt* mutant indeed has a partial replicative defect within the macrophages as compared to parent, which may reflect its pH sensitivity.

Other LTs may have a similar pH dependency. For example, the Mlt38 enzyme from *E. coli* has been shown to harbor maximal enzymatic activity in buffers between pH 4.0 and 4.5 (Ursinus and Holtje, 1994). Furthermore, a recent study showed a *mltB* mutant in *Acinetobacter baumannii* had significantly decreased survival under acidic conditions that was partially restored by complementation (Crépin et al., 2018). We suspect the Slt protein may play an important role during infection of host cells, as we observed a significant decrease in intracellular growth of the Fn mutant as compared to the parent. The study by Crépin et al. (2018) also observed increased sensitivity of the *mltB* mutant to polymyxin B. In contrast, our results showed an increase in resistance to polymyxin B for the Fn *slt* mutant. Generally, Gram-positive bacteria exhibit polymyxin B resistance due to their thick peptidoglycan wall; it is possible that build-up of peptidoglycan in the periplasmic space of cells lacking Slt activity is responsible for this phenotype (Poirel et al., 2017).

Scanning electron microscopy studies of Fn *slt* showed extensive outer membrane surface projections, often connecting neighboring cells, predominantly at pH 6.2. These surface projections have been previously described as outer membrane tubes (OMT), produced in addition to outer membrane vesicles (OMV), during growth of *F. novicida* to early stationary phase in rich medium (McCaig et al., 2013). Initial discovery and characterization of OMVs from *F. novicida* were reported by Pierson et al. (2011); the authors demonstrated OMVs contained numerous virulence-associated proteins and that they could provide significant protection against intranasal *Francisella* challenge. McCaig et al. (2013) further showed that OMTs trigger inflammatory cytokine release from murine macrophages, and are produced during infection of macrophages. These studies further suggest at the vaccine potential of a Fn *slt* mutant that overproduces OMTs able to elicit an effective immune response. A more recent study found that production of OMTs was triggered by cysteine deprivation in rich medium, indicating a role for OMTs in metabolism as well as in virulence (Sampath et al., 2018). Interestingly, *F. novicida* transposon mutants exhibiting hyper-vesiculation had interruptions in genes involved in cell division and peptidoglycan modification, such as *ftsK* and carboxypeptidases like *dacD*. However, this study did not identify *slt* as a hyper-vesiculating mutant, possibly because acidic pH conditions were not tested.

### Role of Slt in Virulence of *F. novicida*

Our experiments demonstrate that Slt is a critical virulence factor in *F. novicida*. The *slt* mutant exhibited decreased replication in macrophages and was significantly attenuated during intranasal infection of mice. Several studies have investigated the impact of lytic transglycosylase enzymes on virulence of bacteria including *Brucella abortus, Edwardsiella tarda, N. gonorrhoeae*, and *A. baumannii*. Deletion of a putative lytic transglycosylase in *B. abortus*, homologous to MltB, resulted in reduced survival in murine macrophages and in mice (Bao et al., 2017). In *E. tarda*, a Gram-negative bacterium pathogenic to fish, deletion of *mltA* reduced virulence in a zebrafish model while overexpression of *mltA* increased virulence (Liu et al., 2012). The most well-characterized LT enzymes, in regards to their effect on virulence and host immune response, are those of *N. gonorrhoeae*. Multiple studies emphasize the importance of lytic transglycosylases in releasing peptidoglycan fragments which trigger inflammation and cell damage, and alter host cell signaling pathways (Schaub et al., 2016; Chan and Dillard, 2017; Knilans et al., 2017). Interestingly, a recent study showed that a *mltB* mutant of *A. baumannii* was significantly reduced in its ability to adhere to alveolar epithelial cells and colonize the lung during mouse infection, likely due to the drastic decrease in surface pili (Crépin et al., 2018). Indeed, lytic transglycosylases have been deemed “space-making enzymes” and are necessary for the incorporation of structures like flagella, pili, and secretion systems that contribute to virulence. For further information, an excellent review by Koraimann (2003) discusses specialized LTs and their contribution to pathogenic structures. Since *Francisella* contains Type IV and VI secretion systems, it would be interesting to see how LT enzymes may contribute to their incorporation and pathogenicity. The relationship between cell wall modifying enzymes, cell physiology, and virulence is only a newly expanding area of study that requires more attention, especially given the implications in antimicrobial resistance, drug design, and inhibitors.

### Immune Response/Vaccine Potential

Generation of an effective vaccine against tularemia is critical, as there is currently no FDA approved vaccine available. Several subunit and live attenuated vaccines have been tested. Whelan et al. (2018) recently showed that a subunit vaccine, comprising *Francisella* LPS and the FTT_0814 protein delivered within glucan particles, was able to induce protection against Ft Schu S4 challenge in the Fischer 344 rat model. However, other subunit vaccines based on LPS, carbohydrates, and other surface antigens were unable to provide complete protection, and it is thought that both humoral and cell-mediated immune responses are required to achieve protective immunity (Pechous et al., 2009). Numerous live vaccines based on mutants of *F. novicida, F. tularensis*, and LVS have been explored, including those with mutations in FPI, metabolism, purine biosynthesis, and LPS biosynthesis genes (Pechous et al., 2009; Jia and Horwitz, 2018). One of the most promising vaccines tested is the Δ*clpB* mutant, which contains a deletion in the heat shock gene *clpB*, developed by Conlan et al. (2010) in the Ft Schu S4 background. The authors showed that the Δ*clpB* mutant was more attenuated than LVS, and that intradermal vaccination could protect against subsequent respiratory challenge with Ft Schu S4 (Conlan et al., 2010; Twine et al., 2012). Interestingly, *F. novicida ΔiglD* was shown to be protective against challenge with the fully virulent *F. tularensis* Schu S4 strain in both Fisher344 rats and cynomolgous macaques, indicating the potential for a *F. novicida*-based live vaccine (Chu et al., 2014). A *Francisella* vaccine derived from an LT mutant or other peptidoglycan enzyme mutant has not been described in the literature. However, a lytic transglycosylase mutant, Δ22915, derived from *B. abortus* has been shown to generate a robust immune response in mice and was protective against infection with the WT strain (Bao et al., 2017).

Our initial vaccination studies show that even low doses of the Fn *slt* mutant were able to protect against high challenge doses of the Fn parent. Moreover, our immune response analyses suggested the potential of using the highly attenuated Fn *slt* mutant as a vaccine strain to protect against tularemia. Vaccination of mice with Fn *slt* produced a robust Th17-type (IL-17a, and IL-22), Th1-type (TNF-α, IFN-γ, and IL-2), and Th2-type cytokine (IL-4, IL-5, IL-10, and IL-13) responses, although the fold change in cytokine secretion relative to naïve mice biased the Th17- and Th2-type cytokine profiles. Furthermore, in spite of Th1- vs. Th2-type cytokine fold change differences, the levels of Th1- and Th17-type cytokine responses based on actual concentrations was significantly induced by the Fn *slt* vaccine. Th1 response along with robust production of IFN-γ have previously been established to be necessary for protection against *F. tularensis* infection (Anthony et al., 1989; Sunagar et al., 2018). Based on the marked levels of IL-17a and IL-22, the contribution of the Th17 response also appears to be critical in protection against Fn *slt* challenge (Woolard et al., 2008; Lin et al., 2009; Markel et al., 2010). But, despite Ft Schu S4 re-stimulation inducing a similar Th17 response, the mice were not protected from Ft Schu S4 challenge. Other groups reported similar findings, such that the superfluous role of IL17a in conferring protection was dependent on whether the challenge strain was an attenuated or a virulent *F. tularensis* subspecies (Skyberg et al., 2013). It is plausible that the reduced secretion of the anti-inflammatory cytokines, IL-10 and IL-4, in Ft Schu S4 relative to Fn *slt* challenged mice restricted adequate control of the Th17 responses which resulted in excessive bystander damage and transitioned from a protective to a pathological immune state (Gu et al., 2008; Heo et al., 2010; Slight et al., 2013). Of course, IL-10 is also a potent suppressor of macrophage functions and down-regulator of Th1-type responses and in concert with IL-4 is able to further suppress other essential protective responses (Gazzinelli et al., 1992; Oswald et al., 1992). The balance between Ft Schu S4 clearance and immunopathology are not just Th1 and IFN-γ dependent, but are also in part dictated by other cytokines such as IL-10 and IL-4.

Overall antibody response was poor against both irradiated Fn *slt* and Ft Schu S4, although the antibody titers against both strains within all three vaccine doses remained similar. There is a noticeable, though not significant, increase in antibody titers with increasing vaccine dose against both Fn *slt* and Ft Schu S4. Although, the antibody response against both strains was similar, it may only contribute to controlling infection in the low virulence strain of *F. novicida*, which is consistent with other studies (Fulop et al., 2001). It is evident that a stronger antibody response or antibodies against other epitopes are necessary to control Ft Schu S4 infection or that protective immunity is more reliant on a stronger cell mediated immune response. Often, the level of IgG1 antibodies correlate with an overall Th2 immune response profile while that of IgG2a antibodies is indicative of an overall Th1 profile. Surprisingly, given the propensity of BALB/c mice to mount a more Th2-like immune response, an IgG2a isotype was predominant, relative to IgG1 in serum (Watanabe et al., 2004). Although, different pathogens and routes of immunization and challenge have been shown to stimulate different Th cell subsets that deviate from the canonical Th2-biased profile in BALB/c mice (Garcia-Pelayo et al., 2015). Specifically, there appears to be a strong induction of Th1 response in BALB/c mice in the lungs and spleen. Furthermore, a prominent antibody response seems to be superfluous in protection against Ft Schu S4 challenge in out-bred Swiss Webster mice while both CD4+ and CD8+ T-cell responses are indispensable. A homologous Fn *slt* or heterologous (e.g., LPS and LVS) prime-boost vaccination approach may improve both cellular and humoral protective responses (Cole et al., 2009). Overall, our results indicate that pursuing such a vaccine strategy with the *slt* mutant strain under the conditions described here, would not provide protection against Ft Schu S4. Perhaps a different vaccination route and/or additional doses of the mutant strain could provide protection against challenge.

### Implications for Enzyme Targeting

As we and others show that Slt is important for virulence or essential for growth in *Francisella*, this enzyme would be an ideal candidate for targeting by inhibitors. While the crystal structure of Slt from *Francisella* has not been reported, the Slt70 homolog in *E. coli* has been crystallized in complex with a 1,6-anhydromuropeptide substrate. The structure consists of three domains, the U-shaped U-domain, catalytic C-domain, and linker or L-domain, which are primarily dominated by alpha helices (van Asselt et al., 1999). Importantly, the catalytic domain of Slt from *F. tularensis* harbors a conserved Glu478 residue, known to be necessary for enzymatic activity in *E. coli* and other organisms, which may serve as an ideal target for inhibitors (van Asselt et al., 1999). Additionally, combining cell wall enzyme inhibitors with cell wall-targeting antibiotics is thought to help overcome β-lactam resistance (Johnson et al., 2013). Current work in our laboratory is focused on targeting the Slt enzyme of the fully virulent *F. tularensis* Schu S4 strain for broad-spectrum inhibitors.

## Data Availability

The raw data supporting the conclusions of this manuscript will be made available by the authors, without undue reservation, to any qualified researcher.

## Ethics Statement

This study was carried out in accordance with the recommendations of the Guide for the Care and Use of Laboratory Animals, National Research Council, 2011 and the United States Army of Medical Research Institute of Infectious Diseases Institutional Animal Care and Use Committee. The protocol was approved by the United States Army of Medical Research Institute of Infectious Diseases under an Institutional Animal Care and Use Committee in compliance with the Animal Welfare Act, PHS Policy, and other Federal statutes and regulations relating to animals and experiments involving animals. The facility where this research was conducted is accredited by the Association for Assessment and Accreditation of Laboratory Animal Care.

## Author Contributions

BB, SB, JC, FB, and JB contributed conception and design of the study. BB, SB, JC, SR, RT, CC, CK, MH, JS, JW, KK, FB, and JB participated in the experimentation and acquisition of data. BB, SB, JC, JW, and JB were involved in the analysis or interpretation of data for the work. BB, SB, and JB wrote the manuscript. All authors contributed to manuscript revision, read and approved the submitted version.

## Conflict of Interest Statement

The authors declare that the research was conducted in the absence of any commercial or financial relationships that could be construed as a potential conflict of interest.

## Footnotes

1 https://www.cdc.gov/tularemia/statistics/index.html

## References

[B1] Anthony L. S. Ghadirian E. Nestel F. P. Kongshavn P. A. (1989). The requirement for gamma interferon in resistance of mice to experimental tularemia. *Microb. Pathog.* 7 421–428. 10.1016/0882-4010(89)90022-3 2516219

[B2] Bao Y. Tian M. Li P. Liu J. Ding C. Yu S. (2017). Characterization of *Brucella abortus* mutant strain Δ22915, a potential vaccine candidate. *Vet. Res.* 48:17. 10.1186/s13567-017-0422-9 28376905 PMC5381064

[B3] Ben Nasr A. Haithcoat J. Masterson J. E. Gunn J. S. Eaves-Pyles T. Klimpel G. R. (2006). Critical role for serum opsonins and complement receptors CR3 (CD11b/CD18) and CR4 (CD11c/CD18) in phagocytosis of *Francisella tularensis* by human dendritic cells (DC): uptake of *Francisella* leads to activation of immature DC and intracellular survival of the bacteria. *J. Leukoc. Biol.* 80 774–786. 10.1189/jlb.1205755 16857732

[B4] Boisset S. Caspar Y. Sutera V. Maurin M. (2014). New therapeutic approaches for treatment of tularaemia: a review. *Front. Cell. Infect. Microbiol.* 4:40 10.3389/fcimb.2014.00040 PMC3975101 24734221

[B5] Bozue J. Cote C. K. Webster W. Bassett A. Tobery S. Little S. (2012). A *Yersinia pestis* YscN ATPase mutant functions as a live attenuated vaccine against bubonic plague in mice. *FEMS Microbiol. Lett.* 332 113–121. 10.1111/j.1574-6968.2012.02583.x 22537022

[B6] Bradburne C. E. Verhoeven A. B. Manyam G. C. Chaudhry S. A. Chang E. L. Thach D. C. (2013). Temporal transcriptional response during infection of type II alveolar epithelial cells with *Francisella tularensis* live vaccine strain (LVS) supports a general host suppression and bacterial uptake by macropinocytosis. *J. Biol. Chem.* 288 10780–10791. 10.1074/jbc.M112.362178 23322778 PMC3624459

[B7] Brunton J. Steele S. Miller C. Lovullo E. Taft-Benz S. Kawula T. (2015). Identifying *Francisella tularensis* genes required for growth in host cells. *Infect. Immun.* 83 3015–3025. 10.1128/iai.00004-15 25987704 PMC4496600

[B8] Chamberlain R. E. (1965). Evaluation of live tularemia vaccine prepared in a chemically defined medium. *Appl. Microbiol.* 13 232–235. 14325885 10.1128/am.13.2.232-235.1965 PMC1058227

[B9] Chan J. M. Dillard J. P. (2017). Attention Seeker: production, modification, and release of inflammatory peptidoglycan fragments in *Neisseria* species. *J. Bacteriol.* 199:e00354-17. 10.1128/jb.00354-17 28674065 PMC5637178

[B10] Chance T. Chua J. Toothman R. G. Ladner J. T. Nuss J. E. Raymond J. L. (2017). A spontaneous mutation in *kdsD*, a biosynthesis gene for 3 Deoxy-D-manno-Octulosonic Acid, occurred in a ciprofloxacin resistant strain of *Francisella tularensis* and caused a high level of attenuation in murine models of tularemia. *PLoS One* 12:e0174106. 10.1371/journal.pone.0174106 28328947 PMC5362203

[B11] Chu P. Cunningham A. L. Yu J.-J. Nguyen J. Q. Barker J. R. Lyons C. R. (2014). Live attenuated *Francisella novicida* vaccine protects against *Francisella tularensis* pulmonary challenge in rats and non-human primates. *PLoS Pathog.* 10:e1004439. 10.1371/journal.ppat.1004439 25340543 PMC4207810

[B12] Cloud K. A. Dillard J. P. (2004). Mutation of a single lytic transglycosylase causes aberrant septation and inhibits cell separation of *Neisseria gonorrhoeae*. *J. Bacteriol.* 186 7811–7814. 10.1128/jb.186.22.7811-7814.2004 15516597 PMC524912

[B13] Cole L. E. Yang Y. Elkins K. L. Fernandez E. T. Qureshi N. Shlomchik M. J. (2009). Antigen-specific B-1a antibodies induced by *Francisella tularensis* LPS provide long-term protection against *F. tularensis* LVS challenge. *Proc. Natl. Acad. Sci. U.S.A.* 106 4343–4348. 10.1073/pnas.0813411106 19251656 PMC2657382

[B14] Conlan J. W. Shen H. Golovliov I. Zingmark C. Oyston P. C. F. Chen W. (2010). Differential ability of novel attenuated targeted deletion mutants of *Francisella tularensis* subspecies *tularensis* strain SCHU S4 to protect mice against aerosol challenge with virulent bacteria: Effects of host background and route of immunization. *Vaccine* 28 1824–1831. 10.1016/j.vaccine.2009.12.001 20018266 PMC2822029

[B15] Crépin S. Ottosen E. N. Peters K. Smith S. N. Himpsl S. D. Vollmer W. (2018). The lytic transglycosylase MltB connects membrane homeostasis and *in vivo* fitness of *Acinetobacter baumannii*. *Mol. Microbiol.* 109 745–762. 10.1111/mmi.14000 29884996 PMC6185781

[B16] Dijkstra B. W. Thunnissen A. M. (1994). ’Holy’ proteins. II: The soluble lytic transglycosylase. *Curr. Opin. Struct. Biol.* 4 810–813. 10.1016/0959-440x(94)90261-5 7712284

[B17] Dik D. A. Marous D. R. Fisher J. F. Mobashery S. (2017). Lytic transglycosylases: concinnity in concision of the bacterial cell wall. *Crit. Rev. Biochem. Mol. Biol.* 52 503–542. 10.1080/10409238.2017.1337705 28644060 PMC6102726

[B18] Drabner B. Guzman C. A. (2001). Elicitation of predictable immune responses by using live bacterial vectors. *Biomol. Eng.* 17 75–82. 10.1016/s1389-0344(00)00072-1 11222981

[B19] Eigelsbach H. T. Downs C. M. (1961). Prophylactic effectiveness of live and killed tularemia vaccines. I. Production of vaccine and evaluation in the white mouse and guinea pig. *J. Immunol.* 87 415–425. 13889609

[B20] Ellis J. Oyston P. C. Green M. Titball R. W. (2002). Tularemia. *Clin. Microbiol. Rev.* 15 631–646. 12364373 10.1128/CMR.15.4.631-646.2002 PMC126859

[B21] Fulop M. Mastroeni P. Green M. Titball R. W. (2001). Role of antibody to lipopolysaccharide in protection against low- and high-virulence strains of *Francisella tularensis*. *Vaccine* 19 4465–4472. 10.1016/s0264-410x(01)00189-x 11483272

[B22] Gallagher L. A. Ramage E. Jacobs M. A. Kaul R. Brittnacher M. Manoil C. (2007). A comprehensive transposon mutant library of *Francisella novicida*, a bioweapon surrogate. *Proc. Natl. Acad. Sci. U.S.A.* 104 1009–1014. 10.1073/pnas.0606713104 17215359 PMC1783355

[B23] Garcia D. L. Dillard J. P. (2008). Mutations in *ampG* or *ampD* affect peptidoglycan fragment release from *Neisseria gonorrhoeae*. *J. Bacteriol.* 190 3799–3807. 10.1128/jb.01194-07 18390650 PMC2395056

[B24] Garcia-Pelayo M. C. Bachy V. S. Kaveh D. A. Hogarth P. J. (2015). BALB/c mice display more enhanced BCG vaccine induced Th1 and Th17 response than C57BL/6 mice but have equivalent protection. *Tuberculosis* 95 48–53. 10.1016/j.tube.2014.10.012 25467292

[B25] Gazzinelli R. T. Oswald I. P. James S. L. Sher A. (1992). IL-10 inhibits parasite killing and nitrogen oxide production by IFN-gamma-activated macrophages. *J. Immunol.* 148 1792–1796. 1541819

[B26] Gestin B. Valade E. Thibault F. Schneider D. Maurin M. (2010). Phenotypic and genetic characterization of macrolide resistance in *Francisella tularensis* subsp. holarctica biovar I. *J. Antimicrob. Chemother.* 65 2359–2367. 10.1093/jac/dkq315 20837574

[B27] Golovliov I. Twine S. M. Shen H. Sjostedt A. Conlan W. (2013). A Delta*clpB* mutant of *Francisella tularensis* subspecies *holarctica* strain, FSC200, is a more effective live vaccine than *F. tularensis* LVS in a mouse respiratory challenge model of tularemia. *PLoS One* 8:e78671. 10.1371/journal.pone.0078671 24236032 PMC3827231

[B28] Gu Y. Yang J. Ouyang X. Liu W. Li H. Yang J. (2008). Interleukin 10 suppresses Th17 cytokines secreted by macrophages and T cells. *Eur. J. Immunol.* 38 1807–1813. 10.1002/eji.200838331 18506885 PMC2733944

[B29] Heidrich C. Ursinus A. Berger J. Schwarz H. Höltje J.-V. (2002). Effects of multiple deletions of murein hydrolases on viability, septum cleavage, and sensitivity to large toxic molecules in *Escherichia coli*. *J. Bacteriol.* 184 6093–6099. 10.1128/JB.184.22.6093-6099.2002 12399477 PMC151956

[B30] Heo Y. J. Joo Y. B. Oh H. J. Park M. K. Heo Y. M. Cho M. L. (2010). IL-10 suppresses Th17 cells and promotes regulatory T cells in the CD4+ T cell population of rheumatoid arthritis patients. *Immunol. Lett.* 127 150–156. 10.1016/j.imlet.2009.10.006 19895848

[B31] Hornick R. B. Eigelsbach H. T. (1966). Aerogenic immunization of man with live Tularemia vaccine. *Bacteriol. Rev.* 30 532–538. 5917334 10.1128/br.30.3.532-538.1966 PMC378235

[B32] Horzempa J. O’Dee D. M. Shanks R. M. Nau G. J. (2010). Francisella tularensis Delta*pyrF* mutants show that replication in nonmacrophages is sufficient for pathogenesis *in vivo*. *Infect. Immun.* 78 2607–2619. 10.1128/IAI.00134-10 20385757 PMC2876533

[B33] Ireland P. M. Bullifent H. L. Senior N. J. Southern S. J. Yang Z. R. Ireland R. E. (2019). Global analysis of genes essential for *Francisella tularensis* Schu S4 growth *in vitro* and for fitness during competitive infection of Fischer 344 rats. *J. Bacteriol.* 201:e00630-18. 10.1128/JB.00630-18 30642993 PMC6416918

[B34] Jia Q. Horwitz M. A. (2018). Live attenuated tularemia vaccines for protection against respiratory challenge with virulent *F. tularensis* subsp. *tularensis*. *Front. Cell. Infect. Microbiol.* 8:154. 10.3389/fcimb.2018.00154 29868510 PMC5963219

[B35] Johnson J. W. Fisher J. F. Mobashery S. (2013). Bacterial cell-wall recycling. *Ann. N. Y. Acad. Sci.* 1277 54–75. 10.1111/j.1749-6632.2012.06813.x 23163477 PMC3556187

[B36] Jones R. M. Nicas M. Hubbard A. Sylvester M. D. Reingold A. (2005). The infectious dose of *Francisella tularensis* (Tularemia). *Appl. Biosaf.* 10 227–239. 10.1177/153567600501000405

[B37] Kingry L. C. Petersen J. M. (2014). Comparative review of *Francisella tularensis* and *Francisella novicida*. *Front. Cell. Infect. Microbiol.* 4:35 10.3389/fcimb.2014.00035 PMC3952080 24660164

[B38] Knilans K. J. Hackett K. T. Anderson J. E. Weng C. Dillard J. P. Duncan J. A. (2017). *Neisseria gonorrhoeae* lytic transglycosylases LtgA and LtgD reduce host innate immune signaling through TLR2 and NOD2. *ACS Infect. Dis.* 3 624–633. 10.1021/acsinfecdis.6b00088 28585815 PMC5839173

[B39] Koraimann G. (2003). Lytic transglycosylases in macromolecular transport systems of Gram-negative bacteria. *Cell. Mol. Life Sci.* 60 2371–2388. 10.1007/s00018-003-3056-1 14625683 PMC11138577

[B40] Law H. T. Lin A. E. Kim Y. Quach B. Nano F. E. Guttman J. A. (2011). *Francisella tularensis* uses cholesterol and clathrin-based endocytic mechanisms to invade hepatocytes. *Sci. Rep.* 1:192. 10.1038/srep00192 22355707 PMC3240981

[B41] Lin Y. Ritchea S. Logar A. Slight S. Messmer M. Rangel-Moreno J. (2009). Interleukin-17 is required for T helper 1 cell immunity and host resistance to the intracellular pathogen *Francisella tularensis*. *Immunity* 31 799–810. 10.1016/j.immuni.2009.08.025 19853481 PMC2789998

[B42] Liu W. Dong N. Zhang X. H. (2012). Overexpression of *mltA* in *Edwardsiella tarda* reduces resistance to antibiotics and enhances lethality in zebra fish. *J. Appl. Microbiol.* 112 1075–1085. 10.1111/j.1365-2672.2012.05291.x 22443589

[B43] Liu Y. Breukink E. (2016). The membrane steps of bacterial cell wall synthesis as antibiotic targets. *Antibiotics* 5:28. 10.3390/antibiotics5030028 27571111 PMC5039524

[B44] Loveless B. M. Yermakova A. Christensen D. R. Kondig J. P. Heine H. S. III Wasieloski L. P. (2010). Identification of ciprofloxacin resistance by SimpleProbe, High Resolution Melt and Pyrosequencing nucleic acid analysis in biothreat agents: *Bacillus anthracis, Yersinia pestis* and *Francisella tularensis*. *Mol. Cell. Probes* 24 154–160. 10.1016/j.mcp.2010.01.003 20100564

[B45] LoVullo E. D. Sherrill L. A. Pavelka M. S. Jr. (2009). Improved shuttle vectors for *Francisella tularensis* genetics. *FEMS Microbiol. Lett.* 291 95–102. 10.1111/j.1574-6968.2008.01440.x 19067747 PMC2704062

[B46] LoVullo E. D. Sherrill L. A. Perez L. L. Pavelka M. S. Jr. (2006). Genetic tools for highly pathogenic *Francisella tularensis* subsp. *tularensis*. *Microbiology* 152(Pt. 11) 3425–3435. 10.1099/mic.0.29121-0 17074911

[B47] Markel G. Bar-Haim E. Zahavy E. Cohen H. Cohen O. Shafferman A. (2010). The involvement of IL-17A in the murine response to sub-lethal inhalational infection with *Francisella tularensis*. *PLoS One* 5:e11176. 10.1371/journal.pone.0011176 20585449 PMC2887844

[B48] McCaig W. D. Koller A. Thanassi D. G. (2013). Production of outer membrane vesicles and outer membrane tubes by *Francisella novicida*. *J. Bacteriol.* 195 1120–1132. 10.1128/jb.02007-12 23264574 PMC3592013

[B49] McCrumb F. R. (1961). Aerosol infection of man with *Pasteurella tularensis*. *Bacteriol. Rev.* 25 262–267. 16350172 10.1128/br.25.3.262-267.1961 PMC441102

[B50] McLendon M. K. Apicella M. A. Allen L. A. (2006). *Francisella tularensis*: taxonomy, genetics, and Immunopathogenesis of a potential agent of biowarfare. *Annu. Rev. Microbiol.* 60 167–185. 10.1146/annurev.micro.60.080805.142126 16704343 PMC1945232

[B51] Melillo A. Sledjeski D. D. Lipski S. Wooten R. M. Basrur V. Lafontaine E. R. (2006). Identification of a *Francisella tularensis* LVS outer membrane protein that confers adherence to A549 human lung cells. *FEMS Microbiol. Lett.* 263 102–108. 10.1111/j.1574-6968.2006.00413.x 16958857

[B52] Oswald I. P. Gazzinelli R. T. Sher A. James S. L. (1992). IL-10 synergizes with IL-4 and transforming growth factor-beta to inhibit macrophage cytotoxic activity. *J. Immunol.* 148 3578–3582. 1588047

[B53] Oyston P. C. Quarry J. E. (2005). Tularemia vaccine: past, present and future. *Antonie Van Leeuwenhoek* 87 277–281. 10.1007/s10482-004-6251-7 15928980

[B54] Pechous R. D. McCarthy T. R. Zahrt T. C. (2009). Working toward the future: insights into *Francisella tularensis* pathogenesis and vaccine development. *Microbiol. Mol. Biol. Rev.* 73 684–711. 10.1128/MMBR.00028-09 19946137 PMC2786580

[B55] Pierson T. Matrakas D. Taylor Y. U. Manyam G. Morozov V. N. Zhou W. (2011). Proteomic characterization and functional analysis of outer membrane vesicles of *Francisella novicida* suggests possible role in virulence and use as a vaccine. *J. Proteome Res.* 10 954–967. 10.1021/pr1009756 21138299

[B56] Poirel L. Jayol A. Nordmann P. (2017). Polymyxins: antibacterial activity, susceptibility testing, and resistance mechanisms encoded by plasmids or chromosomes. *Clin. Microbiol. Rev.* 30 557–596. 10.1128/cmr.00064-16 28275006 PMC5355641

[B57] Qin A. Scott D. W. Rabideau M. M. Moore E. A. Mann B. J. (2011). Requirement of the CXXC motif of novel *Francisella* infectivity potentiator protein B FipB, and FipA in virulence of *F. tularensis subsp. tularensis*. *PLoS One* 6:e24611. 10.1371/journal.pone.0024611 21931773 PMC3169626

[B58] Qin A. Scott D. W. Thompson J. A. Mann B. J. (2009). Identification of an essential *Francisella tularensis* subsp. *tularensis* virulence factor. *Infect. Immun.* 77 152–161. 10.1128/IAI.01113-08 18981253 PMC2612291

[B59] Rodriguez S. A. Yu J. J. Davis G. Arulanandam B. P. Klose K. E. (2008). Targeted inactivation of *Francisella tularensis* genes by group II introns. *Appl. Environ. Microbiol.* 74 2619–2626. 10.1128/AEM.02905-07 18310413 PMC2394887

[B60] Rohmer L. Fong C. Abmayr S. Wasnick M. Larson Freeman T. J. Radey M. (2007). Comparison of *Francisella tularensis* genomes reveals evolutionary events associated with the emergence of human pathogenic strains. *Genome Biol.* 8:R102. 10.1186/gb-2007-8-6-r102 17550600 PMC2394750

[B61] Romeis T. Vollmer W. Holtje J. V. (1993). Characterization of three different lytic transglycosylases in *Escherichia coli*. *FEMS Microbiol. Lett.* 111 141–146. 10.1111/j.1574-6968.1993.tb06376.x 8405923

[B62] Sampath V. McCaig W. D. Thanassi D. G. (2018). Amino acid deprivation and central carbon metabolism regulate the production of outer membrane vesicles and tubes by *Francisella*. *Mol. Microbiol.* 107 523–541. 10.1111/mmi.13897 29240272

[B63] Saslaw S. Eigelsbach H. T. Prior J. A. Wilson H. E. Carhart S. (1961). Tularemia vaccine study. II. Respiratory challenge. *Arch. Intern. Med.* 107 702–714. 13746667 10.1001/archinte.1961.03620050068007

[B64] Schaub R. E. Chan Y. A. Lee M. Hesek D. Mobashery S. Dillard J. P. (2016). Lytic transglycosylases LtgA and LtgD perform distinct roles in remodeling, recycling and releasing peptidoglycan in *Neisseria gonorrhoeae*. *Mol. Microbiol.* 102 865–881. 10.1111/mmi.13496 27608412 PMC5463997

[B65] Scheurwater E. Reid C. W. Clarke A. J. (2008). Lytic transglycosylases: bacterial space-making autolysins. *Int. J. Biochem. Cell Biol.* 40 586–591. 10.1016/j.biocel.2007.03.018 17468031

[B66] Schwartz J. T. Barker J. H. Long M. E. Kaufman J. McCracken J. Allen L. A. (2012). Natural IgM mediates complement-dependent uptake of *Francisella tularensis* by human neutrophils via complement receptors 1 and 3 in nonimmune serum. *J. Immunol.* 189 3064–3077. 10.4049/jimmunol.1200816 22888138 PMC3436988

[B67] Shanson D. C. Singh J. (1981). Effect of adding cysteine to brain-heart infusion broth on the isolation of *Bacteroides fragilis* from experimental blood cultures. *J. Clin. Pathol.* 34 221–223. 10.1136/jcp.34.2.221 7229105 PMC1146458

[B68] Skyberg J. A. Rollins M. F. Samuel J. W. Sutherland M. D. Belisle J. T. Pascual D. W. (2013). Interleukin-17 protects against the *Francisella tularensis* live vaccine strain but not against a virulent *F. tularensis* type A strain. *Infect. Immun.* 81 3099–3105. 10.1128/iai.00203-13 23774604 PMC3754213

[B69] Slight S. R. Monin L. Gopal R. Avery L. Davis M. Cleveland H. (2013). IL-10 restrains IL-17 to limit lung pathology characteristics following pulmonary infection with *Francisella tularensis* live vaccine strain. *Am. J. Pathol.* 183 1397–1404. 10.1016/j.ajpath.2013.07.008 24007881 PMC3814571

[B70] Spidlova P. Stojkova P. Dankova V. Senitkova I. Santic M. Pinkas D. (2018). *Francisella tularensis* D-Ala D-Ala carboxypeptidase DacD is involved in intracellular replication and it is necessary for bacterial cell wall integrity. *Front. Cell. Infect. Microbiol.* 8:111. 10.3389/fcimb.2018.00111 29692981 PMC5903032

[B71] Sunagar R. Kumar S. Namjoshi P. Rosa S. J. Hazlett K. R. O. Gosselin E. J. (2018). Evaluation of an outbred mouse model for *Francisella tularensis* vaccine development and testing. *PLoS One* 13:e0207587. 10.1371/journal.pone.0207587 30533047 PMC6289435

[B72] Sutera V. Levert M. Burmeister W. P. Schneider D. Maurin M. (2014). Evolution toward high-level fluoroquinolone resistance in *Francisella* species. *J. Antimicrob. Chemother.* 69 101–110. 10.1093/jac/dkt321 23963236

[B73] Trevino S. R. Klimko C. P. Reed M. C. Aponte-Cuadrado M. J. Hunter M. Shoe J. L. (2018). Disease progression in mice exposed to low-doses of aerosolized clinical isolates of *Burkholderia pseudomallei*. *PLoS One* 13:e0208277. 10.1371/journal.pone.0208277 30500862 PMC6267979

[B74] Twine S. Shen H. Harris G. Chen W. Sjostedt A. Ryden P. (2012). BALB/c mice, but not C57BL/6 mice immunized with a Δ*clpB* mutant of *Francisella tularensis* subspecies *tularensis* are protected against respiratory challenge with wild-type bacteria: association of protection with post-vaccination and post-challenge immune responses. *Vaccine* 30 3634–3645. 10.1016/j.vaccine.2012.03.036 22484348

[B75] Ursinus A. Holtje J. V. (1994). Purification and properties of a membrane-bound lytic transglycosylase from *Escherichia coli*. *J. Bacteriol.* 176 338–343. 10.1128/jb.176.2.338-343.1994 8288527 PMC205055

[B76] van Asselt E. J. Thunnissen A.-M. Dijkstra B. W. (1999). High resolution crystal structures of the *Escherichia coli* lytic transglycosylase slt70 and its complex with a peptidoglycan fragment. *J. Mol. Biol.* 291 877–898. 10.1006/jmbi.1999.3013 10452894

[B77] Watanabe H. Numata K. Ito T. Takagi K. Matsukawa A. (2004). Innate immune response in Th1- and Th2-dominant mouse strains. *Shock* 22 460–466. 10.1097/01.shk.0000142249.08135.e9 15489639

[B78] Whelan A. O. Flick-Smith H. C. Homan J. Shen Z. T. Carpenter Z. Khoshkenar P. (2018). Protection induced by a *Francisella tularensis* subunit vaccine delivered by glucan particles. *PLoS One* 13:e0200213. 10.1371/journal.pone.0200213 30296254 PMC6175290

[B79] Woolard M. D. Hensley L. L. Kawula T. H. Frelinger J. A. (2008). Respiratory *Francisella tularensis* live vaccine strain infection induces Th17 cells and prostaglandin E2, which inhibits generation of gamma interferon-positive T cells. *Infect. Immun.* 76 2651–2659. 10.1128/IAI.01412-07 18391003 PMC2423094

[B80] Zwietering M. H. Jongenburger I. Rombouts F. M. van ’t Riet K. (1990). Modeling of the bacterial growth curve. *Appl. Environ. Microbiol.* 56 1875–1881. 16348228 10.1128/aem.56.6.1875-1881.1990 PMC184525

