# Supplementary material for: A Francisella novicida Mutant, Lacking the Soluble Lytic Transglycosylase Slt, Exhibits Defects in Both Growth and Virulence
Source: Front Microbiol. 2019 Jun 14;10:1343. doi: 10.3389/fmicb.2019.01343 (PMC6587636; doi:10.3389/fmicb.2019.01343)
Supplement: Supplementary file 1 [file Data_Sheet_1.PDF]

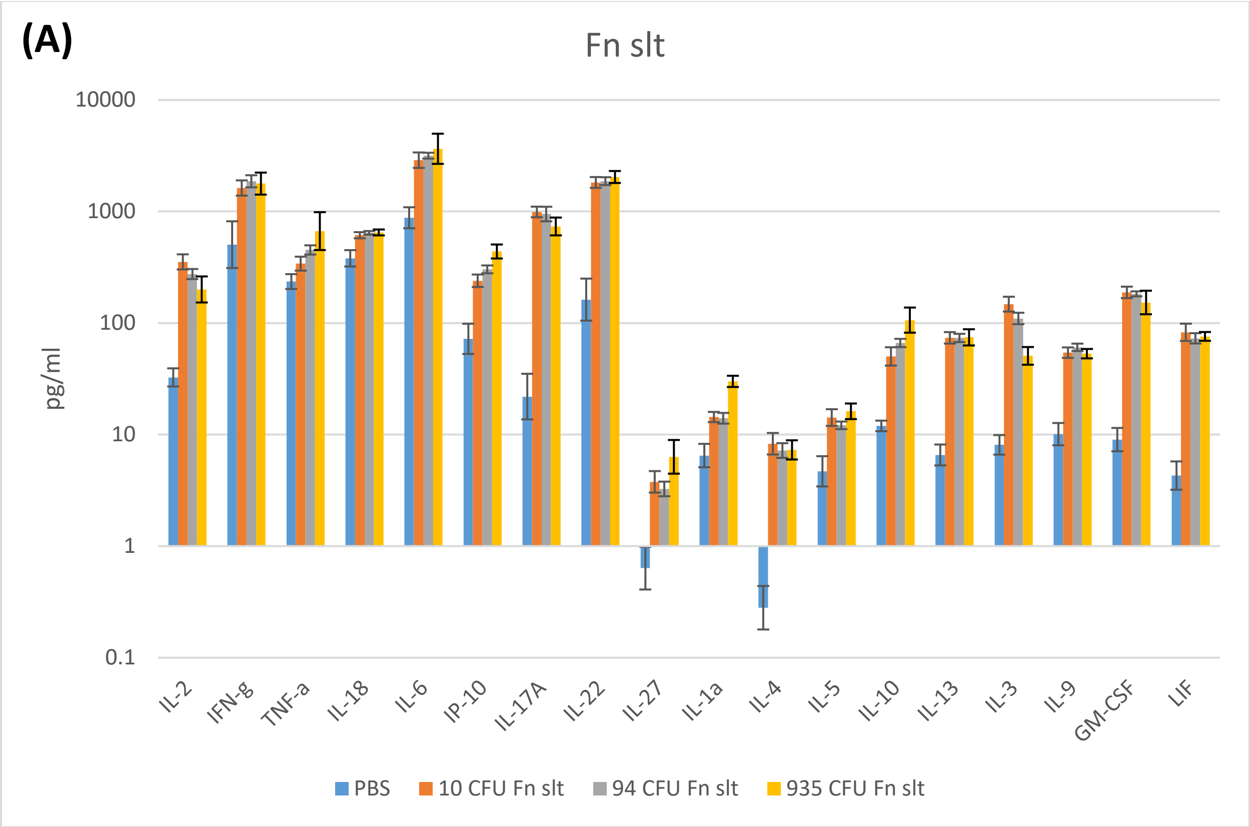

(A)
Fn slt
10000
1000
100
10
1
0.1
pg/ml
IL-2
IFN-g
TNF-a
IL-18
IL-6
IP-10
IL-17A
IL-22
IL-27
IL-1a
IL-4
IL-5
IL-10
IL-13
IL-3
IL-9
GM-CSF
LIF
PBS
10 CFU Fn slt
94 CFU Fn slt
935 CFU Fn slt

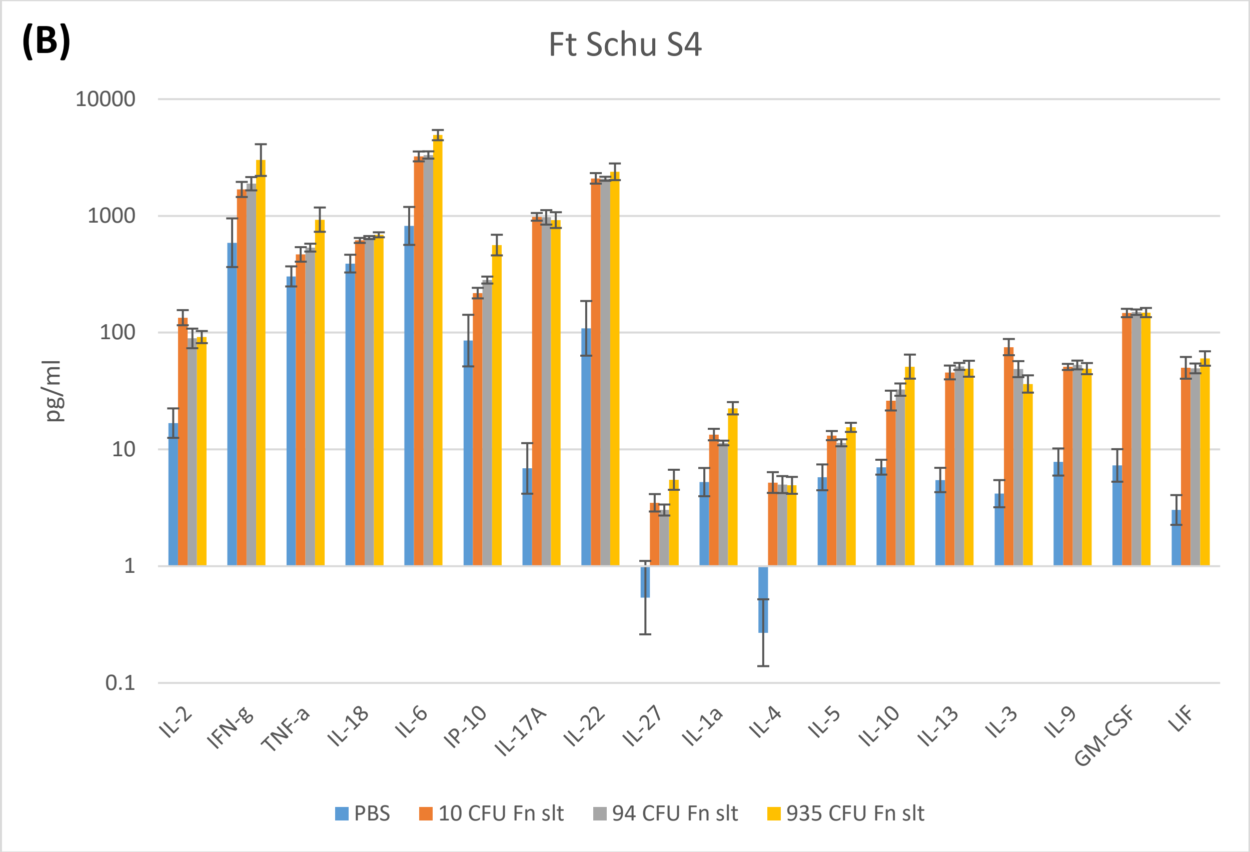

(B)
Ft Schu S4
10000
1000
100
10
1
0.1
pg/ml
IL-2
IFN-g
TNF-a
IL-18
IL-6
IP-10
IL-17A
IL-22
IL-27
IL-1a
IL-4
IL-5
IL-10
IL-13
IL-3
IL-9
GM-CSF
LIF
PBS
10 CFU Fn slt
94 CFU Fn slt
935 CFU Fn slt

Supplementary Figure 1. Cytokine response in splenocytes re-stimulated with Fn *slt* or Ft Schu S4. BALB/c mice were infected intranasally with mock/PBS (n=5), 10 (n= 5), 94 (n=5), and 935 (n=3) CFU Fn *slt* mutant. At day 21 post infection, mice were euthanized and spleens were harvested. Splenocytes (10^6cells) were re-stimulated in the presence of **(A)** irradiated Fn *slt* (5 µg/ml) or **(B)** irradiated *F. tularensis* Schu S4 (5 µg/ml). After 48 hours of re-stimulations, the levels of cytokines were measured by the Luminex bead-based suspension assay. P-values were determined by *t*-test on log10 values compared against PBS only controls. For all 18 analytes shown, P-values of less than 0.05 were obtained and considered significant.
