# Supplementary material for: A Francisella novicida Mutant, Lacking the Soluble Lytic Transglycosylase Slt, Exhibits Defects in Both Growth and Virulence
Source: Front Microbiol. 2019 Jun 14;10:1343. doi: 10.3389/fmicb.2019.01343 (PMC6587636; doi:10.3389/fmicb.2019.01343)
Supplement: Supplementary file 2 [file Data_Sheet_2.PDF]

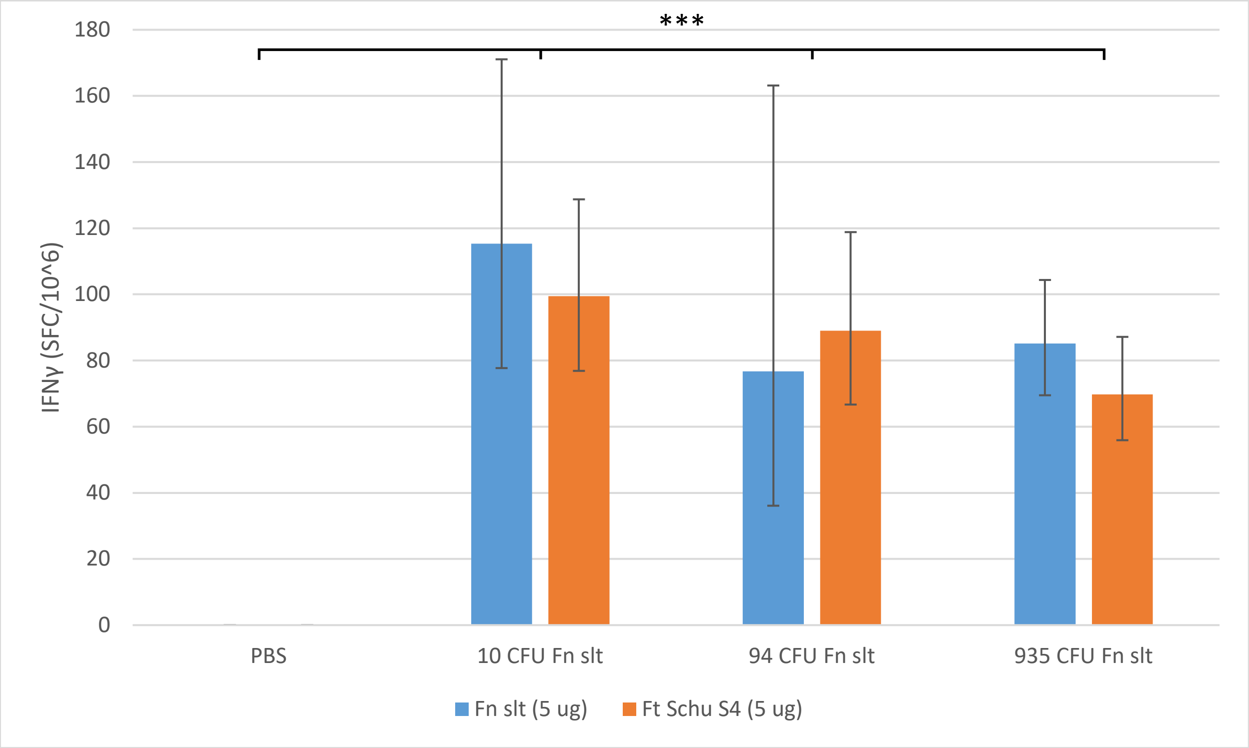

Supplementary Figure 2. Enumeration of specific IFN-γ secreting splenocytes elicited by Fn *slt* or Ft Schu S4. BALB/c mice were infected intranasally with mock/PBS (n=5), 10 (n= 5), 94 (n=5), and 935 (n=3) CFU Fn *slt* mutant. At day 21 post infection, mice were euthanized and spleens were harvested. Splenocytes were re-stimulated in the presence of irradiated Fn *slt* (5 µg/ml) or *F. tularensis* Schu S4 (5 µg/ml). After 24 hours of re-stimulations, the number of discreet IFN-γ secreting cells on the membrane were measured as spot-forming cells (SFC) per 10^6 cells. Statistical significance was determined via *t*-test on log10 values compared against PBS control; ***P<0.0001.
